# Differentially expressed protein and gene analysis revealed the effects of temperature on changes in ascorbic acid metabolism in harvested tea leaves

**DOI:** 10.1038/s41438-018-0070-x

**Published:** 2018-10-01

**Authors:** Hui Li, Zhi-Wei Liu, Zhi-Jun Wu, Yong-Xin Wang, Rui-Min Teng, Jing Zhuang

**Affiliations:** Tea Science Research Institute, College of Horticulture, Nanjing Agricultural University, Nanjing, 210095 China

## Abstract

Tea is an important non-alcoholic beverage worldwide. Tea quality is determined by numerous secondary metabolites in harvested tea leaves, including tea polyphenols, theanine, caffeine, and ascorbic acid (AsA). AsA metabolism in harvested tea leaves is affected by storage and transportation temperature. However, the molecular mechanisms underlying AsA metabolism in harvested tea leaves exposed to different storage and transportation temperature conditions remain unclear. Here we performed RP-HPLC to detect dynamic changes in AsA content in tea leaves subjected to high- (38 °C), low- (4 °C), or room-temperature (25 °C) treatments. The AsA distribution and levels in the treated tea leaves were analyzed using cytological–anatomical characterization methods. The differentially expressed CsAPX1 and CsDHAR2 proteins, which are involved in the AsA recycling pathway, were identified from the corresponding proteomic data using iTRAQ. We also analyzed the expression profiles of 18 genes involved in AsA metabolism, including *CsAPX1* and *CsDHAR2*. AsA was mainly distributed in tea leaf mesophyll cells. High- and low-temperature treatments upregulated the CsAPX1 and CsDHAR2 proteins and induced *CsAPX* and *CsDHAR2* gene expression. These results indicated that the CsAPX1 and CsDHAR2 proteins might have critical roles in AsA recycling in tea leaves. Our results provide a foundation for the in-depth investigation of AsA metabolism in tea leaves during storage and transportation, and they will promote better tea flavor in tea production.

## Tea: storage temperature impacts vitamin C metabolism

High and low temperatures induce the expression of enzymes involved in vitamin C metabolism in tea leaves. A research team from Nanjing Agricultural University, China, led by Jing Zhuang collected fresh leaves from 1-year-old tea plants of the Longjing #43 varietal and then stored the leaves for 4 h at 4 °C (cold), 38 °C (hot), or 25 °C (room temperature). They found that absolute levels of vitamin C—an antioxidant molecule also known as ascorbic acid—were highest after storage at the low temperature and lowest after storage at the high temperature. However, the expression levels of genes involved in vitamin C synthesis and breakdown were elevated at both high and low temperatures. The findings could aid the tea industry in developing storage and transportation strategies that optimize leaf quality and flavor.

## Introduction

The tea plant [*Camellia sinensis* (L.) O. Kuntze] is an economically important crop. Its leaves and leaf buds are used to produce tea, one of the most important and widely consumed non-alcoholic beverages worldwide. Data from the Food and Agriculture Organization of the United Nations (FAO) (http://faostat3.fao.org) website indicated that approximately 2 240 594 ha of land in China was used to cultivate tea plants in 2016. Compounds from green tea could help prevent obesity^1^, cardiovascular disease^2,3^, and Alzheimer’s disease^4^.

Ascorbic acid (AsA), known as vitamin C, is present in plants and several animal species^5,6^. AsA is an organic compound with antioxidant properties^7^. In higher vascular plants, AsA has a vital role in physiological regulation, and it could be involved in the response to ozone, pathogen attack, and senescence^8,9^. Given these functions, AsA is an important organic compound in tea plants^10^. Ivanov et al.^11^ demonstrated that AsA from green tea extracts could inhibit atherogenesis. In rats, AsA derived from green tea could help protect against the toxic effects of orally ingested arsenic and improve cellular antioxidative effects^12,13^.

Four AsA biosynthesis pathways were identified in plants. These pathways include the l-galactose (l-Gal), l-gulose, d-galacturonate, and myo-inositol pathways^14–17^. l-galactose is an important precursor in the l-Gal pathway^18,19^. l-gulose and l-gulono-1,4-lactone are the main intermediates in the l-gulose pathway^17,20^. d-galacturonic acid is a key intermediate in the d-galacturonate pathway^14^. The d-glucuronate-mediated catalysis of myo-inositol into myo-inositol oxygenase (MIOX) is the key reaction of the myo-inositol pathway^15,21^. The l-Gal pathway might be the most validated and well-known AsA biosynthetic pathway in many plants^22^. Although the l-Gal pathway has a vital role in AsA biosynthesis in tea plants, other alternative pathways also participate in AsA biosynthesis in tea plants^23^. The l-Gal pathway is the dominant route of AsA biosynthesis in peach^24^, *Arabidopsis*^25^, carrot^26^, and celery^27^.

Abiotic stresses, including adverse temperature conditions, affect the distribution and levels of AsA in higher plants^28^. High and low temperatures induce ascorbate peroxidase (*APX*) gene expression in tea plants^29^. Low temperatures induce ascorbate peroxidase 3 (*APX3*) gene expression in *Arabidopsis*^30^. Furthermore, the expression levels of the *APX* gene are regulated by various abiotic stresses like salinity, intense light, and hydrogen peroxide (H_2_O_2_)^31–33^_._ Transgenic tobacco carrying the *APX* gene exhibit enhanced low- or high-temperature stress tolerance^34^. The AsA content of red and green transgenic tomato fruits carrying the dehydroascorbate reductase (*DHAR*) gene was increased, but the AsA content of green transgenic tomato fruit carrying the monodehydroascorbate reductase (*MDHAR*) gene was decreased^35^. Under chilling temperatures, MDHAR activity in tomato fruit was significantly correlated with AsA content^36^. In contrast, monodehydroascorbate reductase 3 activity was negatively related to AsA content in tomato leaves^37^. Transgenic tobacco plants carrying the *DHAR* gene from *Arabidopsis* showed improved Al-stress tolerance^38^. In addition, *Arabidopsis* overexpressing the rice *DHAR* gene showed enhanced tolerance to salt stress^39^.

In recent years, with the development of the tea industry and the expansion of tea cultivation areas, traditional artificial tea manufacturers have been replaced by machines. Large numbers of tea leaves are needed to meet the ever-increasing demands of the tea industry. Consequently, the storage and transportation of tea leaves have become important concerns in tea production. Transferring fresh tea leaves from the tea farm to the factory for processing takes several hours. Tea leaves are always stored away from heat to maintain freshness and prevent mold growth. Tea leaves are usually stored and transported under low- (4 °C) and room-temperature (25 °C) conditions. However, the high water content (~70%) in fresh tea leaves sometimes causes the internal temperature of tea leaves to rapidly increase to 38 °C during storage and transportation at room temperature. AsA is one of the important secondary metabolites in tea leaves. Different temperatures during the process of storage and transportation can affect the quality of fresh tea leaves, the flavor of processed tea, and AsA metabolism. The effects of different temperature conditions on the molecular mechanisms underlying the secondary metabolite production of tea leaves during storage and transportation remain unclear. AsA metabolism in tea leaves during storage and transportation requires study.

“Longjing 43” is a typical tea plant cultivar widely cultivated for its high and stable production, and it is also used as a model for genetic and breeding research on tea plants^40–42^. This study was designed to investigate the effects of different temperatures on AsA metabolism in harvested tea leaves of “Longjing 43”. We performed an integrated transcriptome analysis and used isobaric tags for relative and absolute quantitation (iTRAQ) analysis to reveal the effects of high- (38 °C), low- (4 °C), and room-temperature (25 °C) treatments on the potential molecular mechanisms of AsA metabolism in Longjing 43 tea leaves. The differentially expressed proteins (DEPs) involved in AsA metabolism and the expression profiles of 18 genes related to AsA metabolism were identified. Furthermore, we discussed the morphological and anatomical characteristics and AsA distribution and level of tea leaves subjected to different temperature conditions. An understanding of the AsA levels and suitable storage and transportation conditions of tea leaves may provide guidance to the tea industry.

## Materials and methods

### Plant materials, growth conditions, and temperature treatments

One-year-old tea plant cuttings (*C*. *sinensis* cv. “Longjing 43”) were cultivated in an artificial climate chamber at Nanjing Agricultural University, Nanjing, China (32°02′ N, 118°50′ E). The soil type used for tea plant growth contained a mixture of peat, vermiculite, and perlite (3:2:1, v/v). Thirty clonally propagated tea plants were grown under an artificial climate chamber condition (25 °C for 16 h during daytime, 18 °C for 8 h during the dark, 150 μmol/m^2^/s light intensity, and 75% relative humidity). Tea leaves were collected as previously described^41^, laid flat, and wilted for approximately 4 h under different temperature treatments (4, 38, and 25 °C) on 27 September 2016. One bud and two leaves were collected from each sample. The sample for each treatment was picked from 10 tea plants, mixed, and divided into three biological replicates. Samples wilted for 4 h under 25 °C were used as the control. The tea leaves were used for RNA isolation and AsA content determination.

### Dynamic changes in AsA levels

The oxalic acid method was performed as previously described for the determination of AsA levels in tea leaves^23^. Briefly, each fresh sample (200 mg) was homogenized in 4 mL of 1.0% (w/v) oxalic acid (24094A, Shanghai, Adamas, China) and centrifuged at 10 000 × *g* for 10 min at 4 °C. The reaction solution was used to assay AsA content using reversed-phase high-performance liquid chromatography (RP-HPLC). A Shimadzu LC-20A series (Shimadzu Co., Kyoto, Japan) with a Hedera ODS-2 C18 analytical column (250 mm × 4.6 mm i.d., 5 μm nominal particle size) was used for chromatographic separation analysis at 254 nm. Methyl alcohol (MS#1922-801, TEDIA, Susong, China) was used as mobile phase A, and 0.1% (w/v) oxalic acid was used as mobile phase B. The ratio of mobile phase A to B was 5%:95%. Finally, the AsA level was determined by injecting 20 μL of filtrate into the RP-HPLC system and recorded as mg/100 g fresh weight (FW).

### Cytological–anatomical structure and AsA distribution analysis

Paraffin-embedded sections of tea leaves were analyzed using a previously described method with some modifications to investigate the cytological–anatomical structures and AsA distribution in tea leaves^43^. Briefly, leaves, including major veins, were cut into sections with dimensions of 2.5 mm × 2.5 mm. Subsequently, the excised leaf sections were fixed in FAA solution (ethanol:formalin:acetic acid, 90:5:5, v:v:v) (Servicebio, Wuhan, China). After 20 min of deparaffinization with xylene (20641F, Shanghai, Adamas, China) and 40 min of dehydration with ethanol (73537S, Shanghai, Adamas, China), the sections were stained for 1–2 h with 1% safranin O solution (59222C, Shanghai, Adamas, China) and washed with tap water. The sections were dehydrated with 50% ethanol solution (v/v) for 1 min, 70% ethanol solution (v/v) for 1 min, and 80% ethanol solution (v/v) for 1 min. Subsequently, the samples were stained with 0.5% fast green solution (39722B, Shanghai, Adamas, China) for 30–60 s. The sections were dried at 60 °C and washed for 5 min with xylene. Finally, the sections were sealed with neutral balsam for further analysis.

To observe the distribution of AsA in tea leaves, the acidic–alcoholic AgNO_3_ method was used in accordance with the procedure described by Chinoy^44^. In this method, samples were immersed in a mixture solution composed of distilled water:ethanol:acetic acid (29:66:10, v:v:v). Leaf samples were vacuumized for 1 h in 5% AgNO_3_ (N#A4769, Shanghai, Adamas, China) (silver nitrate:mixture solution, w/v) and stored for 24 h at 4 °C. Subsequently, excess residue was washed off for 20 min with 70% ethanol solution containing 5% ammonium acetate (72564A, Shanghai, Adamas, China). Finally, samples were analyzed as paraffin sections without safranin O/fast green staining.

### Total RNA isolation and cDNA reverse transcription

Total RNA was extracted from three samples in accordance with the instructions included with the Quick RNA isolation Kit (Aidlab, Beijing, China). RNA extraction was performed with RNase-free DNaseI (TaKaRa, Dalian, China) to eliminate genomic DNA contamination. The extracted RNA concentration of each sample was calculated using a Nanodrop 2000 spectrophotometer (Thermo Scientific, Wilmington, DE, USA). The first-strand cDNA of each sample was synthesized in accordance with the instructions included with the PrimeScript RT reagent kit (TaKaRa, Dalian, China). The synthetized cDNA of each sample was diluted 18 times and used as template for quantitative real-time PCR (qRT-PCR) amplification. The experiments were repeated as three independent biological samples.

### Protein extraction, labeling, liquid chromatography-tandem mass spectrometry analysis and data analysis

Lysis solution [9 M urea, 4% 3-[(3-cholamidopropyl) dimethylammonio]-1-propane sulfonate, 1% dithiothreitol, and 1% immobilized pH gradient buffer (GE Healthcare)] was used to extract protein from each sample. The mixture was centrifuged at 15 000 rpm for 15 min after 1 h of incubation at 30 °C. Dissolution buffer (AB Sciex, Foster City, CA, USA) was used to dissolve the total protein from each sample, and each sample was labeled using an iTRAQ Reagent-8 plex Multiplex Kit (AB Sciex). The labeled sample was separated by liquid chromatography (LC) with an Eksigent nanoLC-Ultra 2D system (AB SCIEX).

LC fractions were analyzed with a Triple TOF 5600 mass spectrometer (AB SCIEX) under the following conditions: ion spray voltage, 2.5 kV; nebulizer gas pressure, 5 PSI; curtain gas pressure, 30 PSI; and interface heater temperature, 150 °C. The information-dependent acquisition mode was applied for 35 product ion scans (2^+^ to 5^+^) above a threshold ion count of 150 in the mass spectrometry survey scan. The dynamic exclusion duration was 18 s. The data for iTRAQ protein were analyzed using Protein Pilot Software Version 4.0 against the database Uniprot_grape.

### Database search for DEPs, qRT-PCR, and protein–protein interaction analysis

The DEPs involved in the AsA recycling pathway in tea leaves subjected to different temperature treatments were identified using iTRAQ-based quantitative proteomics^41^. The protein–protein interaction (PPI) prediction was performed using the search tool for the retrieval of interacting genes/proteins (STRING) (Version 10.0, http://string-db.org/)^45^. Additionally, the interaction networks of proteins and AsA were constructed using STITCH (search tool for interacting chemicals; http://stitch.embl.de/)^46^.

### Gene expression analysis through qRT-PCR

The 18 AsA metabolism-related genes were identified by searching the known amino-acid sequences from carrot against tea plant genomic or transcriptomic database using Bioedit software^26,47–49^. The primers (Table 1) used in the qRT-PCR analysis were designed using Primer Premier 6.0 software. The *CsTBP* gene is a stable reference gene that has been used in the gene expression analysis of tea plants^40^. Here the *CsTBP* gene was used as the reference gene for normalizing the expression levels of AsA metabolism-related genes in different samples. A Bio-Rad iQ5 platform (Bio-Rad, Hercules, CA, USA) system was used for the qRT-PCR analysis, which was performed using SYBR Premix *Ex Taq* (Tli RNaseH Plus; TaKaRa, Dalian, China) in accordance with the manufacturer’s protocol. Each reaction volume of 15 μL contained 7.2 μL of deionized water, 5.5 μL of SYBR Premix *Ex Taq* (Tli RNaseH Plus; TaKaRa, Dalian, China), 1.5 μL of diluted cDNA strands, and 0.4 μL of each primer. The thermal cycling conditions of qRT-PCR were as follows: 95 °C for 30 s; 40 cycles at 95 °C for 5 s; and 55 °C for 25 s. The transcript abundance measurements of each reaction were repeated as three independent biological samples.

**Table 1:** qRT-PCR primer sequences of genes related to AsA biosynthetic and recycling pathways in tea leaves

Name	Forward primer (5ʹ–3ʹ)	Reverse primer (5ʹ–3ʹ)
*CsPMM*	CCACATTATTAGCTTCCTTCTCGTCAC	CCAACAACACCAACTGTAACAACCTT
*CsGGP*	ATCTTCCTTGTACCACAGTGTTATGCT	TGCCTCCTCGTAGTCCTTCTTCC
*CsGME*	AACTACGGAGCATACACCTATGAGAAC	CTAGCAATGTGCGAGGCAATGAATC
*CsGMP*	GAACTCGGTTGAGACCATTGACACTT	CCACTTCACTCACTCCAATAGCCTTG
*CsGPP*	GCTGCTGGTGCTGTGGTAGAAT	CTAGAAGTGACTGCTCCACCTTATCG
*CsGalLDH*	GGCGGCATTGTTCAGGTTGGT	GTCCACAGCGAGCAAGATAGAATAGTT
*CsGalDH*	GAGAGTGACTAGGAGCATTGATGAGAG	CCAAGCGGAAGTCCTGTAATACCAA
*CsPMI*	TCTGCGGTCAATATTCACTCAACTCAT	TGTTCCTTATCTGTCAACTGCCTCAC
*CsPGI1*	CATTGTGAAGAGTCAGCAACCTGTGTA	CGATTGCCAGAGAAGGTCTTGTGAG
*CsPGI2*	CGATGTCGTCAGTGGTAAGATTAAGC	TTATCTTGAGAGGCGGATTATCAGGAG
*CsAPX*	AGCAAGGTCACGAAGCCAACAAT	GCAACAACTCCAGCCAACTGATAGA
*CsMIOX*	CGTCAATCACATCAACCAA	ACTCTCATCCACAACATCAT
*CsGalUR*	GAGCAGCCTCTTGGAGAAGCAAT	ATCACGATGAGCATCAGAACACCAA
*CsAO*	CCAACACCACTCAAGCACTAACAATAC	GAGGATGATACGGCGGTGATGG
*CsDHAR1*	ATGATGGAACCGAGCAAGCATTACT	GACAAGTCCGCAGCAGATACTCTT
*CsDHAR2*	ACCCTCCTCTCTGCCATTCTCC	TTCATCCAGTGCCTTCAACTCATCAA
*CsGR*	ACCCTGATGGCTAATAAGAATGCTGAA	TAGTATGTGCCTTGCCGAGTAGAGT
*CsMDHAR*	AGACTCTCGTTAGTGCTGCTGGA	TCTTCGCCTGAATTGCTTCTACAAGT
*CsTBP*	GGCGGATCAAGTGTTGGAAGGGAG	ACGCTTGGGATTGTATTCGGCATTA

### Statistical analysis

Experiments on water loss, AsA contents, and expression levels of AsA metabolism-related genes were performed with three independent samples. Data were analyzed using the Statistical Package for Social Sciences (SPSS) statistics version 17.0 (SPSS, Inc., Chicago, IL, USA). Differences in the expression levels of AsA metabolism-related genes, water levels, and AsA contents were detected using Duncan’s multiple-range test at a *P* < 0.05 probability level. Data are presented as the mean ± standard deviation.

## Results

### Morphological changes and weight loss exhibited by tea leaves subjected to different temperature treatments

**Fig. 1:**
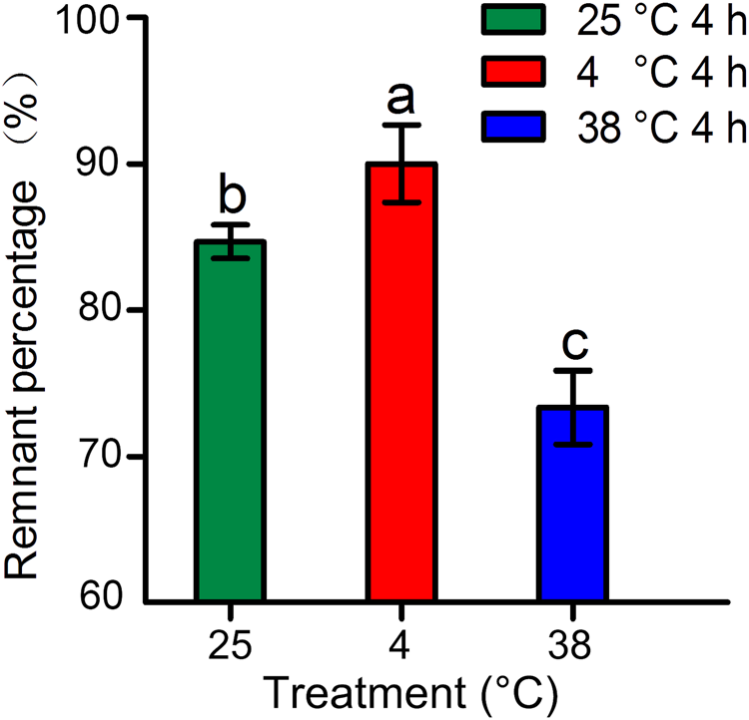
Effects of temperature treatments on the water loss of tea leaves. Error bars represent the standard deviation among three independent replicates. Data are presented as the mean ± SD of three independent replicates. Different lowercase letters indicate significant differences at *P* < 0.05 based on three biological repetitions

The morphological changes and weight losses exhibited by tea leaves subjected to different temperature treatments were investigated. The remnant percentages of tea leaves were 90.01%, 84.67%, and 73.33% at 4, 25, and 38 °C, respectively (Fig. 1).

### AsA levels of tea leaves

**Fig. 2:**
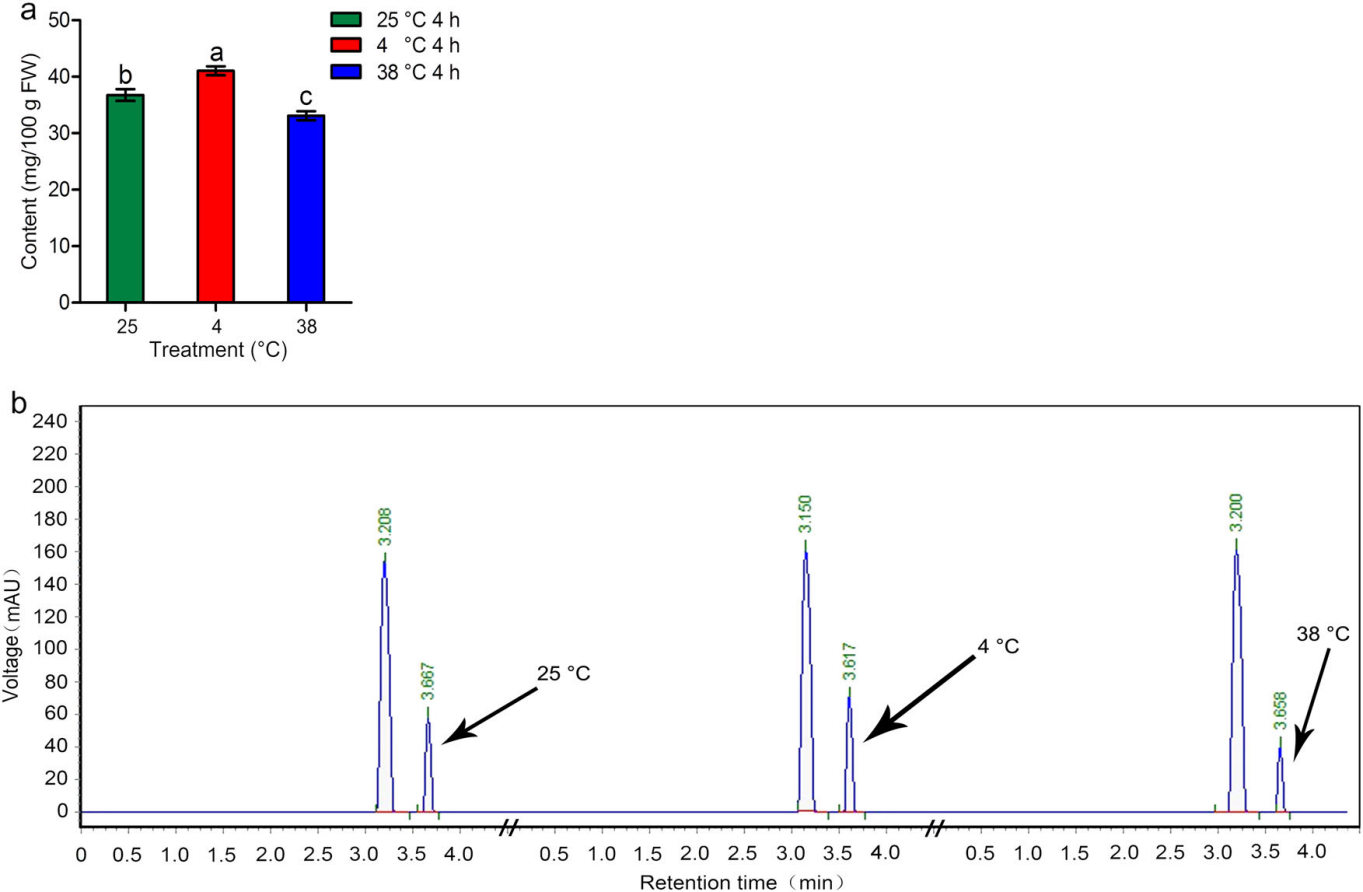
AsA contents and AsA RP-HPLC profiles of tea leaves under different temperature treatments. **a** AsA contents of tea leaves under different temperature treatments. **b** AsA RP-HPLC profiles of tea leaves under different temperature treatments. Error bars represent the standard deviation among three independent replicates. Data for AsA content are presented as the mean ± SD of three independent replicates. Different lowercase letters indicate significant differences at *P* < 0.05 based on three biological repetitions

The AsA levels of tea leaves were measured using RP-HPLC. The standard curve for AsA concentration was constructed prior to sample measurement (Fig. S1). The AsA levels of tea leaves under different temperature treatments differed significantly (Fig. 2a). Figure 2b shows the HPLC profiles of AsA in tea leaves subjected to different temperature treatments. Among the three treatments, the highest AsA level (41.06 mg/100 g) was recorded after 4 °C treatment, and the lowest level (33.09 mg/100 g) was recorded after 38 °C treatment. The AsA level in tea leaves subjected to 25 °C treatment was 36.77 mg/100 g.

**Fig. 3:**
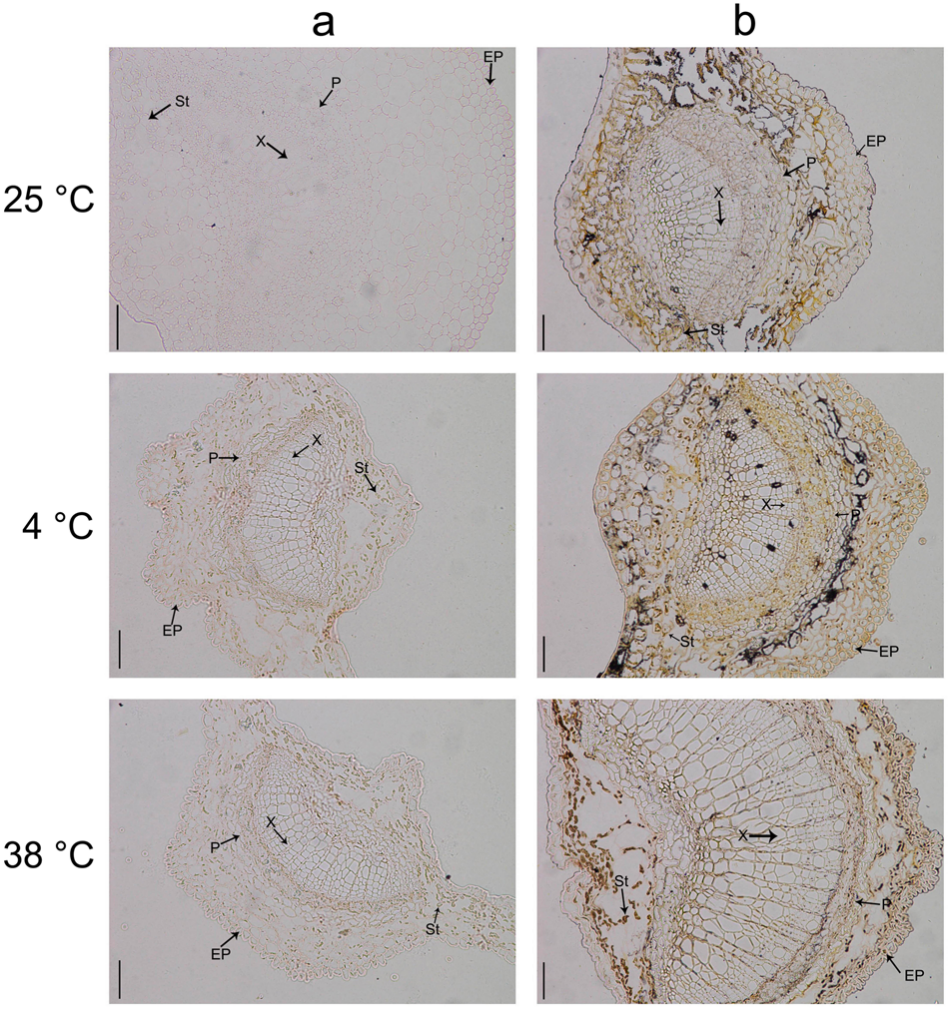
Distribution of AsA in tea leaves under different temperature treatments. **a** Control group. **b** Cool acidic–alcoholic AgNO_3_ treatment group. Epidermis (Ep), spongy tissue (St), phloem (P), and xylem (X) are marked in the figure. The scale bar represents a length of 50 μm

### AsA distribution in tea leaves

Cytological–anatomical structure analysis was performed to elucidate the effects of different temperature treatments on the AsA level and distribution in tea leaves. The bioactive AsA levels in tea leaves significantly changed under high- and low-temperature treatments (Fig. 3a, b). As inferred from the distribution of black spots throughout the leaves, the highest AsA levels were observed in leaves subjected to 4 °C treatment. The lowest AsA content was observed in leaves subjected to 38 °C treatment. AsA was mainly distributed in the spongy tissue of tea leaves that received 4 and 25 °C treatments, and it was rarely distributed in the xylem of tea leaves that received 38 °C treatment.

### Identification of the DEPs in the AsA recycling pathway in tea leaves

**Fig. 4:**
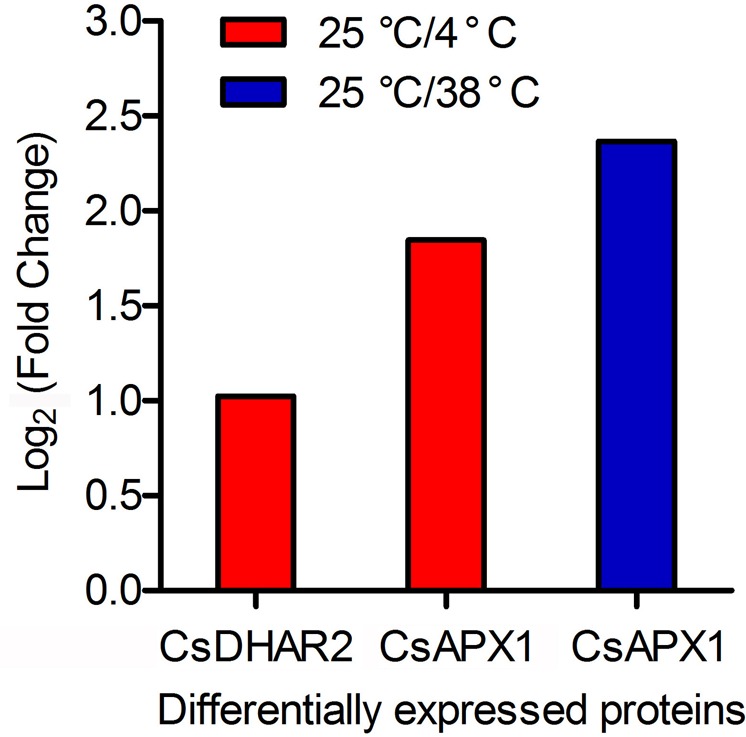
Expression profiles of DEPs in the AsA recycling pathway in tea leaves under different temperature treatments

The DEPs of tea leaves that received different temperature treatments were analyzed using iTRAQ (Fig. 4; Table 2). In this study, two AsA metabolism-related proteins were identified in leaves subjected to 4 °C treatment: CsAPX1 (Table S1) and CsDHAR2 (Table S2). The CsAPX1 protein was also identified in leaves treated with 38 °C (Table S3). These AsA metabolism-related proteins were differentially expressed in tea leaves under different temperature treatments. Under 4 °C treatment, the expression level of CsDHAR2 was lower than CsAPX1.

**Table 2:** DEP information for AsA metabolism-related proteins in tea leaves under high- and low-temperature treatments

Treatment	Protein name	Gene name	ID	Fold change	Log_2_ (fold change)	Identity	*E*-value	Uniprot	Uniprot_URL	Transcription factor
High temperature	Ascorbate peroxidase 1	*CsAPX1*	Q1AFF4	5.1522878	2.365213183	84.8	2.0E-148	Q05431	http://www.uniprot.org/uniprot/Q05431	FALSE
Low temperature	Dehydroascorbate reductase 2	*CsDHAR2*	A9UFY0	2.032356635	1.023153586	71.36	8.0E-100	Q9FRL8	http://www.uniprot.org/uniprot/Q9FRL8	FALSE
	Ascorbate peroxidase 1	*CsAPX1*	Q1AFF4	3.597494194	1.846992359	84.8	2.0E-148	Q05431	http://www.uniprot.org/uniprot/Q05431	FALSE

### Gene expression profiles of AsA metabolism-related genes in tea leaves

**Fig. 5:**
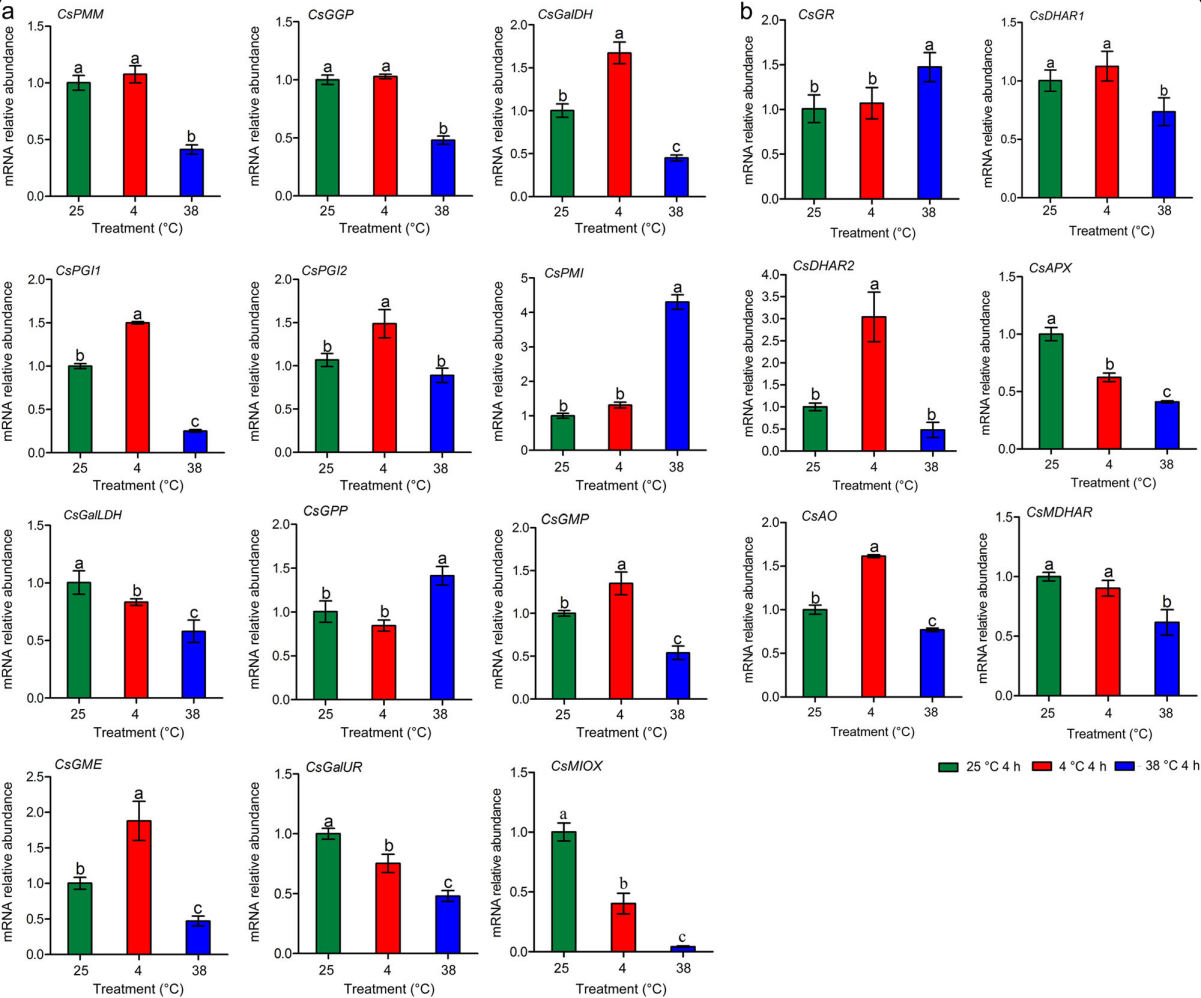
qRT-PCR analysis of the expression levels of genes involved in AsA biosynthesis and recycling pathways in tea leaves under different temperature treatments. **a** Genes involved in the AsA biosynthetic pathway. **b** Genes involved in the AsA recycling pathway. Phosphomannose mutase (*CsPMM*), GDP-l-galactose phosphorylase (*CsGGP*), l-galactose dehydrogenase (*CsGalDH*), phosphoglucose isomerase 1 (*CsPGI1*), phosphoglucose isomerase 2 (*CsPGI2*), phosphomannose isomerase (*CsPMI*), l-galactono-1,4-lactone dehydrogenase (*CsGalLDH*), GDP-d-mannose pyrophosphorylase (*CsGMP*), GDP-d-mannose-3ʹ,5ʹ-epimerase (*CsGME*), l-galactose-1-P phosphatase (*CsGPP*), d-galacturonate reductase (*CsGalUR*), myo-inositol oxygenase (*CsMIOX*), glutathione reductase (*CsGR*), dehydroascorbate reductase 1 (*CsDHAR1*), dehydroascorbate reductase 2 (*CsDHAR2*), ascorbate peroxidase (*CsAPX*), ascorbate oxidase (*CsAO*), and monodehydroascorbate reductase (*CsMDHAR*). Error bars represent the standard deviation among the three independent replicates. Data are presented as the mean ± SD of three independent replicates. Different lowercase letters indicate significant differences at *P* < 0.05 based on three biological replicates

The expression levels of genes involved in AsA biosynthetic and recycling pathways in tea leaves under different temperature treatments were detected to explore the effects of temperature on the expression profiles of the genes of interest (Fig. 5). Genes involved in the AsA biosynthetic and recycling pathways were identified by referring to the tea plant transcriptome database.

The expression levels of five genes (*CsGalDH*, *CsPGI1*, *CsPGI2*, *CsGMP*, and *CsGME*) involved in the AsA biosynthetic pathway were upregulated under low-temperature (4 °C) treatment relative to room-temperature (25 °C) treatment. The expression levels of 10 genes (*CsPMM*, *CsGGP*, *CsGalDH*, *CsPGI1*, *CsPGI2*, *CsGalLDH*, *CsGMP*, *CsGME*, *CsGalUR*, and *CsMIOX*) were downregulated under high-temperature (38 °C) treatment compared to room-temperature treatment. In addition, the expression levels of *CsGPP* and *CsPMI* were significantly upregulated under high-temperature treatment compared to under room-temperature treatment. Among the genes involved in the AsA recycling pathway, the expression levels of four genes (*CsDHAR1*, *CsAPX*, *CsAO*, and *CsMDHAR*) were downregulated under high-temperature treatment, and two genes (*CsDHAR2* and *CsAO*) were upregulated under low-temperature treatment compared to room-temperature treatment. *CsAPX* and *CsMIOX* expression levels were suppressed under high- or low-temperature treatments.

### PPI analysis

**Fig. 6:**
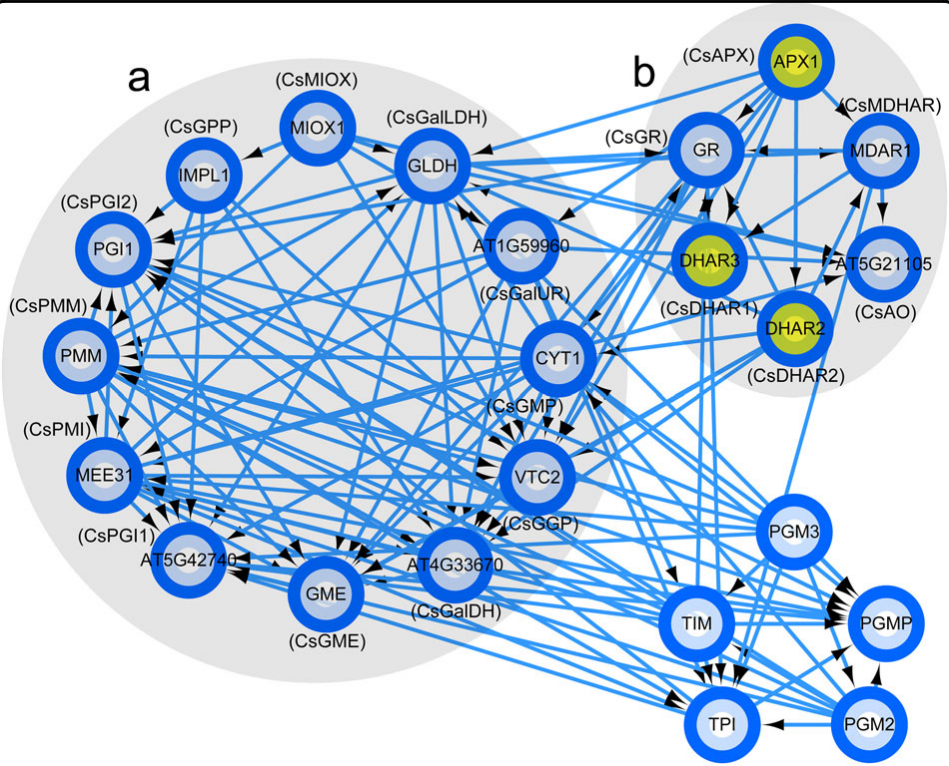
PPI of proteins in the AsA metabolic pathway. **a** Proteins involved in the AsA biosynthetic pathway. **b** Proteins involved in the AsA recycling pathway. Associations among proteins shared by tea plants and *Arabidopsis* jointly contribute to a common function

**Fig. 7:**
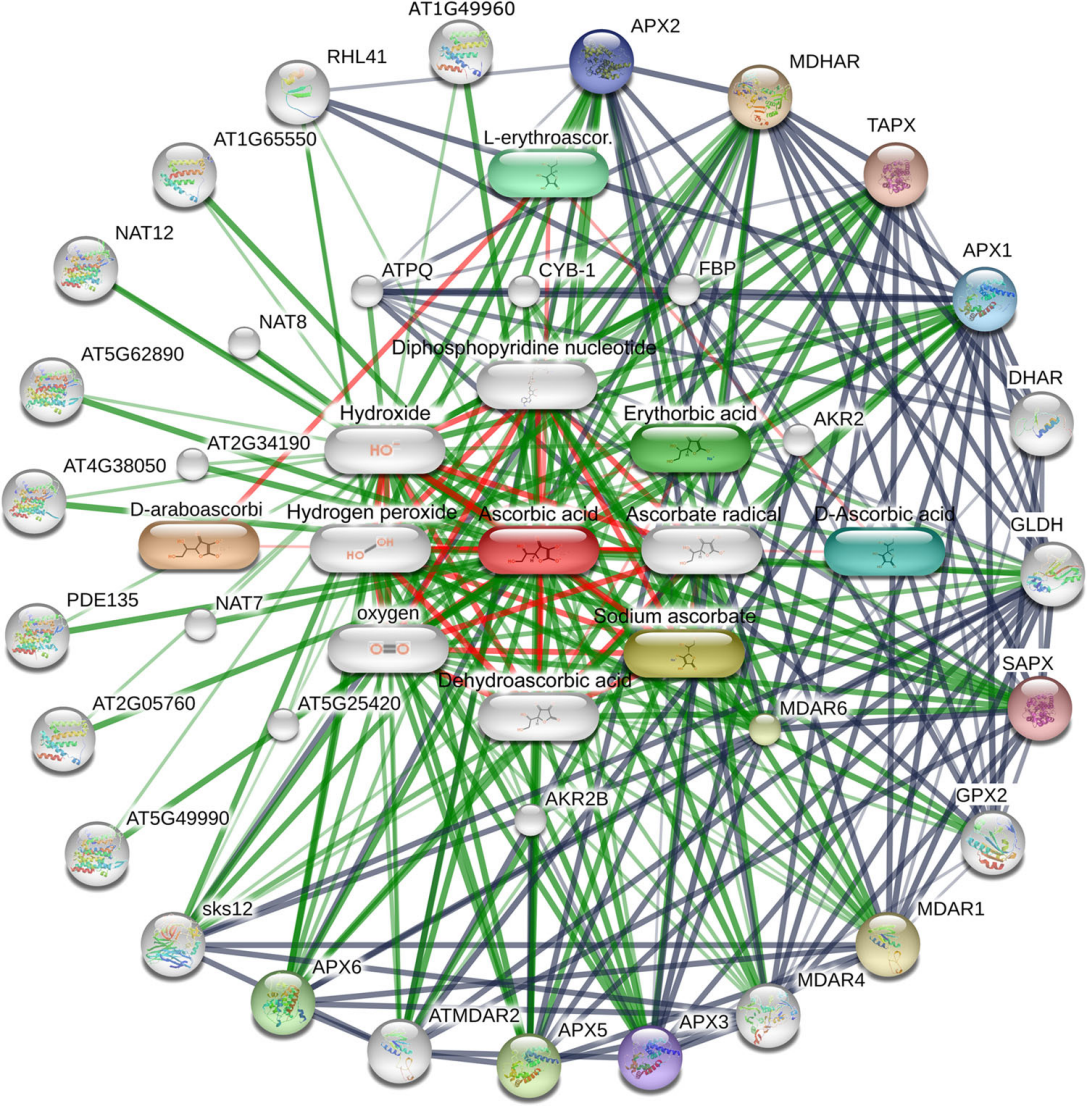
Interaction network of chemicals, proteins, and AsA in *Arabidopsis*. The image presents a comprehensive view. Thick lines represent strong associations. Protein–protein interactions are shown in gray. Chemical–protein interactions are shown in green. Chemical–chemical interactions are shown in red

STRING was used to construct the PPI networks of AsA metabolism DEPs in both *C*. *sinensis* (NCBI Taxonomy ID: 542762) under different temperature treatments and *Arabidopsis* (NCBI Taxonomy ID: 3702). Sequence similarity analysis indicated that AsA metabolism-related proteins in tea plants corresponded to those in *Arabidopsis* *thaliana* (Table S4). STRING was applied to construct a PPI network between the AsA metabolism-related proteins of tea plants and those of *Arabidopsis* (Fig. 6). The results indicated that CsAPX interacts with CsDHAR1 and CsDHAR2. CsDHAR1 and TPI are coexpressed. TPI is a protein involved in gluconeogenesis. In addition, CsDHAR2 and GR are coexpressed. Subsequently, STITCH was used to analyze the known and predicted interaction networks of chemicals, proteins, and AsA (Fig. 7). AsA interacts with some chemical compounds, including erythorbic acid, diphosphopyridine nucleotide, sodium ascorbate, ascorbate radical, dehydroascorbic acid, and hydrogen peroxide.

## Discussion

### Effects of temperature on the morphology and weight loss of tea leaves

High or low temperatures have various effects on plant morphology. For example, low temperature helps prevent the postharvest weight loss of fruits during storage^50^. By contrast, high temperature accelerates the postharvest weight loss of sugarcane cultivars during storage^51^. In this study, we found that different temperature treatments resulted in the postharvest weight loss of tea leaves. Postharvest weight loss often accompanies postharvest physiological deterioration in sweet cherry^52^. Temperature elevations during storage might promote the physiological deterioration of broccoli buds^53^. High temperature could cause cellular dehydration in plants^54^. The cell membrane mediates the selective transport of ions and organic molecules from the external environment^55^ and receives temperature stress signals^56^. It also maintains the shape of the cell by anchoring the cytoskeleton. Cell membrane damage causes cellular dehydration, which might affect weight loss in tea leaves at high temperatures.

### Effects of temperature on AsA level and distribution in tea leaves

External factors like sunlight, temperature, relative humidity, oxidative stress, and pollution influence AsA distribution and levels in plants^57,58^. In this study, the effects of different temperature conditions on the AsA level and distribution in tea leaves were detected and analyzed. AsA levels in tea leaves under low-temperature treatment are higher than those in tea leaves under room- or high-temperature treatment. AsA levels in sea buckthorn leaves and stems decreased with increasing temperature^59^. Temperature stress could affect the contents of antioxidant and lipid peroxidation enzymes^60^. Because AsA is an antioxidant, its levels could also be influenced by temperature stress^9^. Cold stress increases the AsA content of pepper (*Capsicum annuum* L.)^61^. A similar finding was observed for *Cistus* *clusii* under field conditions and a Mediterranean climate^62^.

Considerable evidence from different studies suggested that the AsA distribution differs across plant species. For example, AsA is mainly distributed in the mesocarp, septum, and loculi of ripening tomato fruits^63^ and in the vascular tissues and peel of apple fruits^64^. In plants, AsA is negligibly distributed in vacuoles and is mainly located in the cytoplasm^65,66^. However, in this study, we found that AsA mainly localizes in the mesophyll cells in tea leaves.

### Effects of temperature on the expression levels of AsA metabolism-related genes in tea leaves

Gene expression in most plants is affected by abiotic stress, including high or low temperature stress^67,68^. In most plants, the gene expression levels involved in AsA metabolism are related to abiotic stress^69,70^. Although AsA metabolism-related genes were detected and identified in tea plants on the basis of transcriptome data and the expression of AsA metabolism-related genes in response to temperature stress was analyzed^23,29^, the expression patterns of AsA metabolism-related genes in tea leaves under different temperature stresses remain unclear.

In apple leaves, the expression levels of *APX*, *DHAR*, and *GR* decreased after 4 h of continuous high-temperature treatment^71^. In this study, we found that the expression levels of the *CsAPX* and *CsDHAR1* genes decreased and *CsGR* increased under treatment with 38 °C. Exposing tomatoes after harvest to temperatures from 12 to 3 °C for 56 h inhibits *GME1* expression^28^. Similarly, we found that exposing tea leaves to temperatures of 25 to 38 °C for 4 h decreased *CsGME* expression. In *A*. *Actinidia eriantha*. *GGP* expression was inhibited after heat treatment (42 °C) relative to normal conditions (25 °C)^72^. Here *CsGGP* expression was reduced after treatment at 38 °C compared to treatment at 25 °C.

In harvested tomato, *GPP* is the only AsA biosynthetic gene that was affected by 4 °C treatment. Its expression level peaked after 3 h of treatment and decreased to virtually undetectable levels after 1 h of treatment at 40 °C. High temperature induced *CsGPP* expression in the tea plant, and *CsGPP* transcript abundance was negatively correlated with AsA levels in tea leaves. *GPP* expression was closely associated with AsA levels during tomato ripening^73^. GPP is an enzyme that converts l-galactose-1-P to l-ascorbate. Shade significantly induced *GPP1* expression in ripe red tomato fruits. *GPP1* expression was correlated with AsA levels in tomato leaves and fruits^74^. These findings implied that *CsGPP* has important associations with AsA levels in tea leaves.

AO is a crucial enzyme in AsA accumulation. Previous studies hinted that the levels of *AO* expression and of AsA are linked. For example, suppressing *AO* expression increased AsA levels in harvested tomato fruits after drought treatment^75^. In this study, we found that *CsAO* expression is positively correlated with AsA levels in tea leaves. Transgenic *Arabidopsis* plants carrying the *CuZnSOD* and *APX* genes showed improved abiotic stress tolerance^76^. In tea leaves, *CsAPX* expression was higher at 25 °C than at 4 °C. *APX* mRNA abundance in harvested potato tubers was higher at 20 °C than at 5 °C^77^.

### DEPs of proteins related to AsA metabolism-related proteins in tea leaves

iTRAQ technology enhanced the precision and reliability of the quantitative analysis of human, animal, and plant proteins^78–80^. The proteomics approach enabled the functional analysis of proteins related to AsA metabolism in plants^81,82^. iTRAQ-based quantitative proteomics analysis showed that drought treatment decreased the expression levels of APX and AO proteins in the leaves of cultivated tobacco^83^. iTRAQ analysis indicated that APX activity decreased from 0 to 12 h but increased from 12 to 96 h during postharvest physiological deterioration^84^. In addition, metabolic proteome analysis showed that the protein abundance of APX1 decreased in the embryo-surrounding tissues of wheat in response to high salt stress^85^. The protein abundance of SlGalLDH in the young leaves and harvested fruit of four transgenic tomato lines was lower than in the young leaves and harvested fruit of control plants^86^. Protein gel blot analysis illustrated that the positive protein signals of LetAPX in four transgenic tomato lines were stronger than in wild-type lines^87^. Proteomic analysis has revealed that APX1 plays a major role in response to drought and heat stress in *Arabidopsis*^88^. The VTC2 protein appears to exhibit a dual function in green tomato tissues^73^. The VTC2 protein shares high similarity with the CsGGP protein. These findings suggest that the function of CsGGP protein might be like the VTC2 protein. iTRAQ-based analysis identified that APX3 and DHAR proteins were expressed during the early or late postharvest physiological deterioration of *M*. *esculenta* at 27 °C^84^.

In this study, iTRAQ-based analysis indicated that CsAPX1 and CsDHAR2 proteins were expressed in tea leaves after 4 h of 4 °C treatment and that the CsAPX1 protein was expressed in tea leaves after 4 h of treatment at 38 °C. Treatment at 38 and 4 °C upregulated CsAPX1 and CsDHAR2 protein expression. Our findings implied that CsAPX1 and CsDHAR2 proteins were involved in AsA recycling pathways in tea leaves. Previous studies also showed that the DHAR1 protein localizes to plant peroxisomes and chloroplasts^89,90^. The APX protein localizes to the cytosol, chloroplast, and peroxisomes^91^. AsA content and distribution are closely associated with chloroplasts^92,93^. In the AsA-GSH cycle of chloroplasts, APX can scavenge H_2_O_2_, DHAR, and MDHAR, and it can catalyze the regeneration of AsA^94,95^. Consequently, these results indicated that CsAPX1 and CsDHAR2 proteins could be involved in the AsA recycling pathway in tea leaves subjected to different temperature treatments.

## Conclusions

**Fig. 8:**
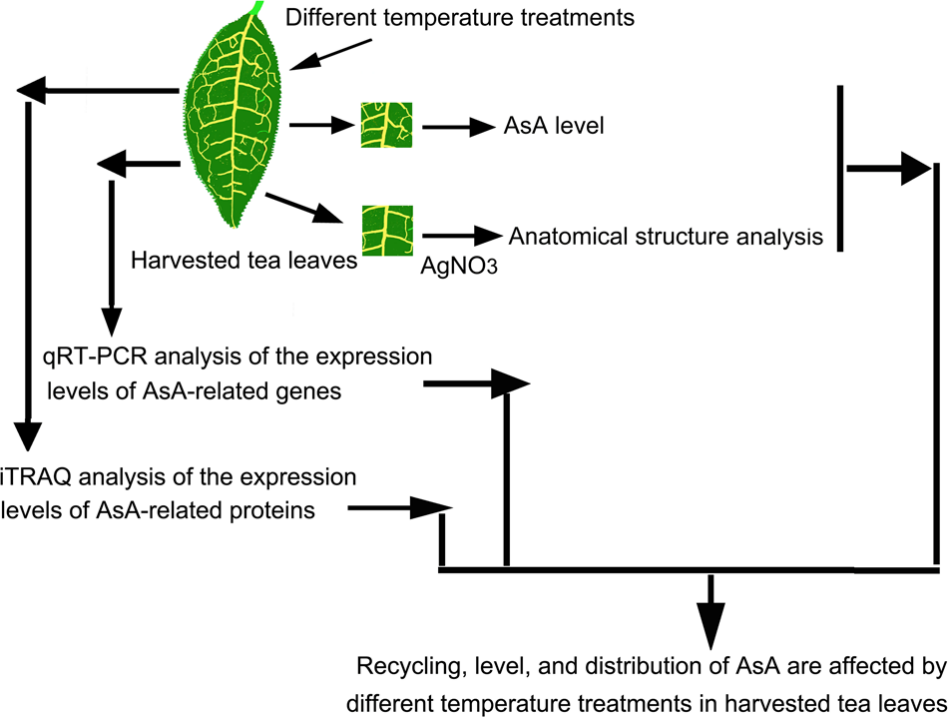
Flow chart of the effects of different temperature treatments on AsA in tea leaves

Numerous studies on the regulatory mechanisms underlying the postharvest quality of various crops were conducted. This study investigated the effects of different temperature treatments on AsA metabolism in tea leaves (Fig. 8). AsA content and distribution in tea leaves subjected to different temperature treatments were investigated. qRT-PCR and iTRAQ were utilized to analyze the expression patterns of proteins and genes related to the AsA metabolic pathway. The results indicated that AsA is mainly distributed in mesophyll cells in tea leaves. High or low temperatures modulate the expression levels of the *CsAPX* and *CsDHAR2* genes. iTRAQ revealed that high and low temperatures upregulated CsAPX1 and CsDHAR2 protein expression. Therefore, CsAPX1 and CsDHAR2 proteins might play important roles in AsA recycling in tea leaves. The data provided by this study will help provide additional insight on the investigation of AsA levels in tea leaves and provide a foundation for strategies to enhance tea product quality and flavor.

## Electronic supplementary material

Table S1

Table S2

Table S3

Table S4

Figure S1

## References

[1] Huang J (2014). The anti-obesity effects of green tea in human intervention and basic molecular studies. Eur. J. Clin. Nutr..

[2] Lambert JD, Sang S, Yang CS (2007). Biotransformation of green tea polyphenols and the biological activities of those metabolites. Mol. Pharm..

[3] Wolfram S (2007). Effects of green tea and EGCG on cardiovascular and metabolic health. J. Am. Coll. Nutr..

[4] Mandel S, Amit T, Baram O, Youdim MB (2007). Iron dysregulation in Alzheimer’s disease: multimodal brain permeable iron chelating drugs, possessing neuroprotective-neurorescue and amyloid precursor protein-processing regulatory activities as therapeutic agents. Prog. Neurobiol..

[5] Gallie DR (2013). The role of L-ascorbic acid recycling in responding to environmental stress and in promoting plant growth. J. Exp. Bot..

[6] Linster CL, Van SE (2007). Vitamin C. Biosynthesis, recycling and degradation in mammals. FEBS J..

[7] Padayatty SJ (2003). Vitamin C as an antioxidant: evaluation of its role in disease prevention. J. Am. Coll. Nutr..

[8] Wang J, Zhang Z, Huang R (2013). Regulation of ascorbic acid synthesis in plants. Plant Signal. Behav..

[9] Conklin PL, Barth C (2004). Ascorbic acid, a familiar small molecule intertwined in the response of plants to ozone, pathogens, and the onset of senescence. Plant Cell Environ..

[10] Hasegawa N, Niimi N, Odani F (2002). Vitamin C is one of the lipolytic substances in green tea. Phytother. Res..

[11] Ivanov V, Roomi MW, Kalinovsky T, Niedzwiecki A, Rath M (2007). Anti-atherogenic effects of a mixture of ascorbic acid, lysine, proline, arginine, cysteine, and green tea phenolics in human aortic smooth muscle cells. J. Cardiovasc. Pharm..

[12] Sárközi K (2016). Green tea and vitamin C ameliorate some neuro-functional and biochemical signs of arsenic toxicity in rats. Nutr. Neurosci..

[13] Son YR, Park TS, Shim SM (2016). Pharmacokinetics and plasma cellular antioxidative effects of flavanols after oral Intake of green tea formulated with vitamin C and xylitol in healthy subjects. J. Med. Food.

[14] Agius F (2003). Engineering increased vitamin C levels in plants by overexpression of a D-galacturonic acid reductase. Nat. Biotechnol..

[15] Lorence A, Chevone BI, Mendes P, Nessler CL (2004). *myo*-inositol oxygenase offers a possible entry point into plant ascorbate biosynthesis. Plant Physiol..

[16] Wheeler GL, Jones MA, Smirnoff N (1998). The biosynthetic pathway of vitamin C in higher plants. Nature.

[17] Wolucka BA, Montagu MV (2003). GDP-mannose 3′,5′-epimerase forms GDP-L-gulose, a putative intermediate for the de novo biosynthesis of vitamin C in plants. J. Biol. Chem..

[18] Li M, Ma F, Guo C, Liu J (2010). Ascorbic acid formation and profiling of genes expressed in its synthesis and recycling in apple leaves of different ages. Plant Physiol. Biochem..

[19] Davey MW (1999). Ascorbate biosynthesis in Arabidopsis cell suspension culture. Plant Physiol..

[20] Wolucka BA, Van Montagu M (2007). The VTC2 cycle and the de novo biosynthesis pathways for vitamin C in plants: an opinion. Phytochemistry.

[21] Zhang W, Gruszewski HA, Chevone BI, Nessler CL (2008). An Arabidopsis purple acid phosphatase with phytase activity increases foliar ascorbate. Plant Physiol..

[22] Linster CL, Clarke SG (2008). L-Ascorbate biosynthesis in higher plants: the role of VTC2. Trends Plant Sci..

[23] Li H (2017). Transcriptomic analysis of the biosynthesis, recycling, and distribution of ascorbic acid during leaf development in tea plant (*Camellia sinensis* (L.) O. Kuntze). Sci. Rep..

[24] Imai T, Ban Y, Terakami S, Yamamoto T, Moriguchi T (2009). L-Ascorbate biosynthesis in peach: cloning of six L-galactose pathway-related genes and their expression during peach fruit development. Physiol. Plant..

[25] Dowdle J, Ishikawa T, Gatzek S, Rolinski S, Smirnoff N (2007). Two genes in *Arabidopsis thaliana* encoding GDP-L-galactose phosphorylase are required for ascorbate biosynthesis and seedling viability. Plant J..

[26] Wang GL (2015). Regulation of ascorbic acid biosynthesis and recycling during root development in carrot (*Daucus carota* L). Plant Physiol. Biochem..

[27] Huang W (2016). Transcriptional profiling of genes involved in ascorbic acid biosynthesis, recycling, and degradation during three leaf developmental stages in celery. Mol. Genet. Genomics.

[28] Massot C (2013). High temperature inhibits ascorbate recycling and light stimulation of the ascorbate pool in tomato despite increased expression of biosynthesis genes. PLoS ONE.

[29] Li H, Huang W, Wang G, Wu Z, Zhuang J (2016). Expression profile analysis of ascorbic acid-related genes in response to temperature stress in the tea plant, *Camellia sinensis* (L) O. Kuntze. Genet. Mol. Res..

[30] Zhang H, Wang J, Nickel U, Allen RD, Goodman HM (1997). Cloning and expression of an Arabidopsis gene encoding a putative peroxisomal ascorbate peroxidase. Plant Mol. Biol..

[31] Bonifacio A (2011). Role of peroxidases in the compensation of cytosolic ascorbate peroxidase knockdown in rice plants under abiotic stress. Plant Cell Environ..

[32] Fryer MJ (2003). Control of ascorbate peroxidase 2 expression by hydrogen peroxide and leaf water status during excess light stress reveals a functional organisation of Arabidopsis leaves. Plant J..

[33] Shi WM, Muramoto Y, Ueda A, Takabe T (2001). Cloning of peroxisomal ascorbate peroxidase gene from barley and enhanced thermotolerance by overexpressing in *Arabidopsis thaliana*. Gene.

[34] Sun WH (2010). Overexpression of tomato tAPX gene in tobacco improves tolerance to high or low temperature stress. Biol. Plant..

[35] Haroldsen VM, Chi-Ham CL, Kulkarni S, Lorence A, Bennett AB (2011). Constitutively expressed DHAR and MDHAR influence fruit, but not foliar ascorbate levels in tomato. Plant Physiol. Biochem..

[36] Stevens R (2008). Tomato fruit ascorbic acid content is linked with monodehydroascorbate reductase activity and tolerance to chilling stress. Plant Cell Environ..

[37] Gest N, Garchery C, Gautier H, Jimenez A, Stevens R (2013). Light-dependent regulation of ascorbate in tomato by a monodehydroascorbate reductase localized in peroxisomes and the cytosol. Plant Biotechnol. J..

[38] Yin L (2010). Overexpression of dehydroascorbate reductase, but not monodehydroascorbate reductase, confers tolerance to aluminum stress in transgenic tobacco. Planta.

[39] Ushimaru T (2006). Transgenic Arabidopsis plants expressing the rice dehydroascorbate reductase gene are resistant to salt stress. Plant Physiol..

[40] Wu ZJ, Tian C, Jiang Q, Li XH, Zhuang J (2016). Selection of suitable reference genes for qRT-PCR normalization during leaf development and hormonal stimuli in tea plant (*Camellia sinensis*). Sci. Rep..

[41] Wu ZJ, Ma HY, Zhuang J (2017). iTRAQ-based proteomics monitors the withering dynamics in postharvest leaves of tea plant (*Camellia sinensis*). Mol. Genet. Genomics.

[42] Liu ZW (2017). CsGOGAT is important in dynamic changes of theanine content in post-harvest tea plant leaves under different temperature and shading spreadings. J. Agric. Food Chem..

[43] Wang GL (2015). Morphological characteristics, anatomical structure, and gene expression: novel insights into gibberellin biosynthesis and perception during carrot growth and development. Hortic. Res..

[44] Chinoy NJ (1969). On the specificity of the alcoholic, acidic silver nitrate reagent for the histochemical localization of ascorbic acid. Histochemie.

[45] Szklarczyk D (2015). STRING v10: protein–protein interaction networks, integrated over the tree of life. Nucleic Acids Res..

[46] Kuhn M, von Mering C, Campillos M, Jensen LJ, Bork P (2008). STITCH: interaction networks of chemicals and proteins. Nucleic Acids Res..

[47] Hall TA (1999). BioEdit: a user-friendly biological sequence alignment editor and analysis program for Windows 95/98/NT. Nucleic Acids Symp. Ser..

[48] Wu ZJ, Li XH, Liu ZW, Xu ZS, Zhuang J (2014). De novo assembly and transcriptome characterization: novel insights into catechins biosynthesis in *Camellia sinensis*. BMC Plant. Biol..

[49] Wei C (2018). Draft genome sequence of *Camellia sinensis* var. sinensis provides insights into the evolution of the tea genome and tea quality. Proc. Natl Acad. Sci. USA.

[50] Lee TC, Zhong PJ, Chang PT (2015). The effects of preharvest shading and postharvest storage temperatures on the quality of ‘Ponkan’ (*Citrus reticulata* Blanco) mandarin fruits. Sci. Hortic..

[51] Vermaab AK, Agarwal AK, Solomon S (2012). Influence of postharvest storage temperature, time, and invertase enzyme activity on sucrose and weight loss in sugarcane. Postharvest Biol. Technol..

[52] Martínez-Romero D (2006). Postharvest sweet cherry quality and safety maintenance by Aloe vera treatment: a new edible coating. Postharvest Biol. Technol..

[53] Zhuang H, Hildebrand DF, Barth MM (1997). Temperature influenced lipid peroxidation and deterioration in broccoli buds during postharvest storage. Postharvest Biol. Technol..

[54] Ristic Z, Williams G, Yang G, Martin B, Fullerton S (1996). Dehydration, damage to cellular membranes, and heat-shock proteins in maize hybrids from different climates. Plant Physiol..

[55] Northcote DH (1968). Structure and function of plant-cell membranes. Br. Med. Bull..

[56] Yadav SK (2010). Cold stress tolerance mechanisms in plants. A review. Agron. Sustain. Dev..

[57] Gest N, Gautier H, Stevens R (2013). Ascorbate as seen through plant evolution: the rise of a successful molecule?. J. Exp. Bot..

[58] Lee SK, Kader AA (2000). Preharvest and postharvest factors influencing vitamin C content of horticultural crops. Postharvest Biol. Technol..

[59] Kanayama Y (2013). Seasonal changes in abiotic stress tolerance and concentrations of tocopherol, sugar, and ascorbic acid in sea buckthorn leaves and stems. Sci. Hortic..

[60] Rosales MA (2006). Antioxidant content and ascorbate metabolism in cherry tomato exocarp in relation to temperature and solar radiation. J. Sci. Food Agric..

[61] Airaki M (2012). Metabolism of reactive oxygen species and reactive nitrogen species in pepper (*Capsicum annuum* L) plants under low temperature stress. Plant Cell Environ..

[62] Munné-Bosch S, Lalueza P (2007). Age-related changes in oxidative stress markers and abscisic acid levels in a drought-tolerant shrub, *Cistus clusii* grown under Mediterranean field conditions. Planta.

[63] Badejo AA (2011). Translocation and the alternative D-galacturonate pathway contribute to increasing the ascorbate level in ripening tomato fruits together with the D-mannose/L-galactose pathway. J. Exp. Bot..

[64] Li MJ, Ma FW, Zhang M, Pu F (2008). Distribution and metabolism of ascorbic acid in apple fruits (*Malus domestica* Borkh cv. Gala). Plant Sci..

[65] Zechmann B (2011). Subcellular distribution of ascorbate in plants. Plant Signal. Behav..

[66] Zechmann B, Stumpe M, Mauch F (2011). Immunocytochemical determination of the subcellular distribution of ascorbate in plants. Planta.

[67] Shinozaki K, Yamaguchi-Shinozaki K, Seki M (2003). Regulatory network of gene expression in the drought and cold stress responses. Curr. Opin. Plant Biol..

[68] Zhuang J, Zhang J, Hou XL, Wang F, Xiong AS (2014). Transcriptomic, proteomic, metabolomic and functional genomic approaches for the study of abiotic stress in vegetable crops. Crit. Rev. Plant Sci..

[69] Tabata K, Takaoka TM (2002). Gene expression of ascorbic acid-related enzymes in tobacco. Phytochemistry.

[70] Tang L (2006). Enhanced tolerance of transgenic potato plants expressing both superoxide dismutase and ascorbate peroxidase in chloroplasts against oxidative stress and high temperature. Plant Cell Rep..

[71] Ma YH (2009). Effects of high temperature on activities and gene expression of enzymes involved in ascorbate-glutathione cycle in apple leaves. Plant Sci..

[72] Li J, Liang D, Li M, Ma F (2013). Light and abiotic stresses regulate the expression of GDP- l -galactose phosphorylase and levels of ascorbic acid in two kiwifruit genotypes via light-responsive and stress-inducible cis -elements in their promoters. Planta.

[73] Ioannidi E (2009). Expression profiling of ascorbic acid-related genes during tomato fruit development and ripening and in response to stress conditions. J. Exp. Bot..

[74] Massot C, Stevens R, Génard M, Longuenesse JJ, Gautier H (2012). Light affects ascorbate content and ascorbate-related gene expression in tomato leaves more than in fruits. Planta.

[75] Zhang YY (2011). Suppressed expression of ascorbate oxidase gene promotes ascorbic acid accumulation in tomato fruit. Plant Mol. Biol. Rep..

[76] Lee SH (2007). Simultaneous overexpression of both CuZn superoxide dismutase and ascorbate peroxidase in transgenic tall fescue plants confers increased tolerance to a wide range of abiotic stresses. Plant Physiol..

[77] Kawakami S, Matsumoto Y, Matsunaga A, Mayama S, Mizuno M (2002). Molecular cloning of ascorbate peroxidase in potato tubers and its response during storage at low temperature. Plant Sci..

[78] Fan J (2011). Comparative iTRAQ proteome and transcriptome analyses of sweet orange infected by “*Candidatus* Liberibacter asiaticus”. Physiol. Plant..

[79] Shetty V (2012). Quantitative immunoproteomics analysis reveals novel MHC class I presented peptides in cisplatin-resistant ovarian cancer cells. Proteomics.

[80] Sui W (2013). Differential proteomic analysis of renal tissue in mesangial proliferative glomerulonephritis using iTRAQ technology. Nephrology.

[81] Aragüez I, Cruzrus E, Botella MAacute, Medinaescobar N, Valpuesta V (2013). Proteomic analysis of strawberry achenes reveals active synthesis and recycling of L-ascorbic acid. Proteomics.

[82] Lópezvidal O (2016). Mitochondrial ascorbate-glutathione cycle and proteomic analysis of carbonylated proteins during tomato (*Solanum lycopersicum*) fruit ripening. Food Chem..

[83] Xie H (2016). iTRAQ-based quantitative proteomic analysis reveals proteomic changes in leaves of cultivated tobacco (*Nicotiana tabacum*) in response to drought stress. Biochem. Biophys. Res. Commun..

[84] Owiti J (2011). iTRAQ-based analysis of changes in the cassava root proteome reveals pathways associated with post-harvest physiological deterioration. Plant J..

[85] Fercha A (2014). Comparative analysis of metabolic proteome variation in ascorbate-primed and unprimed wheat seeds during germination under salt stress. Proteomics.

[86] Alhagdow M (2007). Silencing of the mitochondrial ascorbate synthesizing enzyme l-Galactono-1,4-Lactone dehydrogenase affects plant and fruit development in tomato. Plant Physiol..

[87] Duan M, Feng HL, Wang LY, Li D, Meng QW (2012). Overexpression of thylakoidal ascorbate peroxidase shows enhanced resistance to chilling stress in tomato. Plant Physiol..

[88] Koussevitzky S (2008). Ascorbate peroxidase 1 plays a key role in the response of *Arabidopsis thaliana* to stress combination. J. Biol. Chem..

[89] Tang ZX, Yang HL (2013). Functional divergence and catalytic properties of dehydroascorbate reductase family proteins from Populus tomentosa. Mol. Biol. Rep..

[90] Reumann S (2009). In-depth proteome analysis of Arabidopsis leaf peroxisomes combined with in vivo subcellular targeting verification indicates novel metabolic and regulatory functions of peroxisomes. Plant Physiol..

[91] Murgia I (2004). *Arabidopsis thaliana* plants overexpressing thylakoidal ascorbate peroxidase show increased resistance to Paraquat-induced photooxidative stress and to nitric oxide-induced cell death. Plant J..

[92] Foyer C, Rowell J, Walker D (1983). Measurement of the ascorbate content of spinach leaf protoplasts and chloroplasts during illumination. Planta.

[93] Smirnoff N (2000). Ascorbate biosynthesis and function in photoprotection. Philos. Trans. R. Soc. Lond. B Biol. Sci..

[94] Valero E (2016). Modeling the ascorbate-glutathione cycle in chloroplasts under light/dark conditions. BMC Syst. Biol..

[95] Huang G, Shan C (2018). Lanthanum improves the antioxidant capacity in chloroplast of tomato seedlings through ascorbate-glutathione cycle under salt stress. Sci. Hortic..

