# Supplementary material for: Differentially expressed protein and gene analysis revealed the effects of temperature on changes in ascorbic acid metabolism in harvested tea leaves
Source: Hortic Res. 2018 Oct 1;5:65. doi: 10.1038/s41438-018-0070-x (PMC6165846; doi:10.1038/s41438-018-0070-x)
Supplement: Supplementary file 2 — Table S2 [file 41438_2018_70_MOESM2_ESM.docx]

**Table S2**

**The DEPs information of CsDHAR2 in harvested tea leaves under low temperature treatment**.

| Biological Process | process\|GO:0044712,single-organism catabolic process\|GO:0044267,cellular protein metabolic process\|GO:0044710,single-organism metabolic process\|GO:0044260,cellular macromolecule metabolic process\|GO:0009636,response to toxic substance\|GO:0071704,organic substance metabolic process\|GO:0014070,response to organic cyclic compound\|GO:0090487,secondary metabolite catabolic process\|GO:0019748,secondary metabolic process\|GO:0010583,response to cyclopentenone\|GO:0009987,cellular process\|GO:0006464,cellular protein modification process\|GO:0043412,macromolecule modification\|GO:0036211,protein modification process\|GO:0044763,single-organism cellular process\|GO:0009404,toxin metabolic process\|GO:0009407,toxin catabolic process\|GO:0042221,response to chemical\|GO:0009056,catabolic process\|GO:0010033,response to organic substance\|GO:0055114,oxidation-reduction process\|GO:0044238,primary metabolic process\|GO:0019538,protein metabolic process\|GO:0050896,response to stimulus\|GO:0044237,cellular metabolic process\|GO:0043170,macromolecule metabolic process\|GO:0010731,protein glutathionylation\|GO:0008150,biological_process |
| --- | --- |
| Cell Component: | GO:0005737,cytoplasm\|GO:0005829,cytosol\|GO:0016020,membrane\|GO:0044464,cell part\|GO:0005623,cell\|GO:0005622,intracellular\|GO:0005575,cellular_component\|GO:0044444,cytoplasmic part\|GO:0071944,cell periphery\|GO:0005886,plasma membrane\|GO:0044424,intracellular part |
| Molecular Function | activity\|GO:0042277,peptide binding\|GO:0043167,ion binding\|GO:0016765,transferase activity, transferring alkyl or aryl (other than methyl) groups\|GO:0016740,transferase activity\|GO:0016667,oxidoreductase activity, acting on a sulfur group of donors\|GO:1900750,oligopeptide binding\|GO:0043168,anion binding\|GO:0033218,amide binding\|GO:0016209,antioxidant activity\|GO:0003824,catalytic activity\|GO:0072341,modified amino acid binding\|GO:0016672,oxidoreductase activity, acting on a sulfur group of donors, quinone or similar compound as acceptor\|GO:0015038,glutathione disulfide oxidoreductase activity\|GO:0015036,disulfide oxidoreductase activity\|GO:0015037,peptide disulfide oxidoreductase activity\|GO:0043295,glutathione binding |
| KEGG Pathways |  |

|  |
| --- |
