# Supplementary material for: Differentially expressed protein and gene analysis revealed the effects of temperature on changes in ascorbic acid metabolism in harvested tea leaves
Source: Hortic Res. 2018 Oct 1;5:65. doi: 10.1038/s41438-018-0070-x (PMC6165846; doi:10.1038/s41438-018-0070-x)
Supplement: Supplementary file 3 — Table S3 [file 41438_2018_70_MOESM3_ESM.docx]

**Table S3**

**The DEPs information of CsAPX1 in harvested tea leaves under high temperature treatment**.

| Biological Process | carbohydrate catabolic process\|GO:0043094,cellular metabolic compound salvage\|GO:0051603,proteolysis involved in cellular protein catabolic process\|GO:0010154,fruit development\|GO:0042542,response to hydrogen peroxide\|GO:0050896,response to stimulus\|GO:0009058,biosynthetic process\|GO:0006511,ubiquitin-dependent protein catabolic process\|GO:0044763,single-organism cellular process\|GO:0006753,nucleoside phosphate metabolic process\|GO:0006757,ATP generation from ADP\|GO:0070271,protein complex biogenesis\|GO:0006732,coenzyme metabolic process\|GO:0006733,oxidoreduction coenzyme metabolic process\|GO:0009310,amine catabolic process\|GO:0009791,post-embryonic development\|GO:0009790,embryo development\|GO:0009793,embryo development ending in seed dormancy\|GO:0009416,response to light stimulus\|GO:0043248,proteasome assembly\|GO:0034645,cellular macromolecule biosynthetic process\|GO:0009123,nucleoside monophosphate metabolic process\|GO:0009126,purine nucleoside monophosphate metabolic process\|GO:0009259,ribonucleotide metabolic process\|GO:0044699,single-organism process\|GO:0009719,response to endogenous stimulus\|GO:0006139,nucleobase-containing compound metabolic process\|GO:0080129,proteasome core complex assembly\|GO:0009056,catabolic process\|GO:0042278,purine nucleoside metabolic process\|GO:0046939,nucleotide phosphorylation\|GO:0051179,localization\|GO:0043933,macromolecular complex subunit organization\|GO:0006508,proteolysis\|GO:0032502,developmental process\|GO:0006497,protein lipidation\|GO:0032501,multicellular organismal process\|GO:0048608,reproductive structure development\|GO:0009628,response to abiotic stimulus\|GO:0009987,cellular process\|GO:0019941,modification-dependent protein catabolic process\|GO:0072593,reactive oxygen species metabolic process\|GO:0044106,cellular amine metabolic process\|GO:0006970,response to osmotic stress\|GO:0006972,hyperosmotic response\|GO:0044257,cellular protein catabolic process\|GO:0009408,response to heat\|GO:0009132,nucleoside diphosphate metabolic process\|GO:0009135,purine nucleoside diphosphate metabolic process\|GO:0009642,response to light intensity\|GO:0055086,nucleobase-containing small molecule metabolic process\|GO:0007030,Golgi organization\|GO:0006082,organic acid metabolic process\|GO:0005996,monosaccharide metabolic process\|GO:1901135,carbohydrate derivative metabolic process\|GO:0009059,macromolecule biosynthetic process\|GO:0043170,macromolecule metabolic process\|GO:0006807,nitrogen compound metabolic process\|GO:0048731,system development\|GO:0006006,glucose metabolic process\|GO:0034976,response to endoplasmic reticulum stress\|GO:0009185,ribonucleoside diphosphate metabolic process\|GO:0010035,response to inorganic substance\|GO:0061458,reproductive system development\|GO:0046364,monosaccharide biosynthetic process\|GO:0019752,carboxylic acid metabolic process\|GO:0019693,ribose phosphate metabolic process\|GO:0034622,cellular macromolecular complex assembly\|GO:0006091,generation of precursor metabolites and energy\|GO:0042044,fluid transport\|GO:0043632,modification-dependent macromolecule catabolic process\|GO:0006090,pyruvate metabolic process\|GO:0072524,pyridine-containing compound metabolic process\|GO:0006096,glycolytic process\|GO:0006094,gluconeogenesis\|GO:0071822,protein complex subunit organization\|GO:0009853,photorespiration\|GO:0018377,protein myristoylation\|GO:0009314,response to radiation\|GO:0007275,multicellular organismal development\|GO:0071704,organic substance metabolic process\|GO:0046686,response to cadmium ion\|GO:0044267,cellular protein metabolic process\|GO:0006725,cellular aromatic compound metabolic process\|GO:0006461,protein complex assembly\|GO:0006464,cellular protein modification process\|GO:0072521,purine-containing compound metabolic process\|GO:0044767,single-organism developmental process\|GO:0022414,reproductive process\|GO:0044765,single-organism transport\|GO:0044265,cellular macromolecule catabolic process\|GO:0009117,nucleotide metabolic process\|GO:0009116,nucleoside metabolic process\|GO:0042221,response to chemical\|GO:0009119,ribonucleoside metabolic process\|GO:0043623,cellular protein complex assembly\|GO:0009057,macromolecule catabolic process\|GO:1902578,single-organism localization\|GO:0006996,organelle organization\|GO:0044238,primary metabolic process\|GO:0005975,carbohydrate metabolic process\|GO:0009735,response to cytokinin\|GO:0048856,anatomical structure development\|GO:0006499,N-terminal protein myristoylation\|GO:0006498,N-terminal protein lipidation\|GO:1901657,glycosyl compound metabolic process\|GO:0006796,phosphate-containing compound metabolic process\|GO:0044085,cellular component biogenesis\|GO:0006950,response to stress\|GO:0006793,phosphorus metabolic process\|GO:0019362,pyridine nucleotide metabolic process |
| --- | --- |
| Cell Component: | junction\|GO:0005737,cytoplasm\|GO:0005575,cellular_component\|GO:0009536,plastid\|GO:0009532,plastid stroma\|GO:0005618,cell wall\|GO:0009570,chloroplast stroma\|GO:0016020,membrane\|GO:0044435,plastid part\|GO:0044434,chloroplast part\|GO:0005794,Golgi apparatus\|GO:0030312,external encapsulating structure\|GO:0055044,symplast\|GO:0012505,endomembrane system\|GO:0005911,cell-cell junction\|GO:0005886,plasma membrane\|GO:0009506,plasmodesma\|GO:0009507,chloroplast\|GO:0043231,intracellular membrane-bounded organelle\|GO:0005829,cytosol\|GO:0044464,cell part\|GO:0005623,cell\|GO:0005622,intracellular\|GO:0044446,intracellular organelle part\|GO:0044444,cytoplasmic part\|GO:0044424,intracellular part\|GO:0044422,organelle part |
| Molecular Function | GO:0043169,cation binding\|GO:0046872,metal ion binding\|GO:0020037,heme binding\|GO:0046906,tetrapyrrole binding\|GO:0097159,organic cyclic compound binding\|GO:0016209,antioxidant activity\|GO:0043167,ion binding\|GO:1901363,heterocyclic compound binding\|GO:0003824,catalytic activity\|GO:0003674,molecular_function\|GO:0005488,binding\|GO:0016688,L-ascorbate peroxidase activity\|GO:0016684,oxidoreductase activity, acting on peroxide as acceptor\|GO:0004601,peroxidase activity\|GO:0016491,oxidoreductase activity |
| KEGG Pathways | ath00053,Ascorbate and aldarate metabolism\|ath00480,Glutathione metabolism |

|  |
| --- |
