# Supplementary material for: Differentially expressed protein and gene analysis revealed the effects of temperature on changes in ascorbic acid metabolism in harvested tea leaves
Source: Hortic Res. 2018 Oct 1;5:65. doi: 10.1038/s41438-018-0070-x (PMC6165846; doi:10.1038/s41438-018-0070-x)
Supplement: Supplementary file 4 — Table S4 [file 41438_2018_70_MOESM4_ESM.docx]

**Table S4**

**The proteins in *Arabidopsis thaliana* appear to match the AsA-related proteins in tea plant by STRING**.

| Protein Name | Query Sequence | STRING Protein of *Arabidopsis* | Annotation | Identity | Bitscore |
| --- | --- | --- | --- | --- | --- |
| CsAO | MGGSFFNRILVPSSSADPCSRACRVALLICCCLSVLVESSLGSKTRHFKWEVEYMYWSPDCVEGVVLGINGQFPGPTIRARAGDIVNVELKNKLTTEGVVIHWHGIRQQGTPWADGTASISQCVINPGETFVYRFKVERAGTYFYHGHYGMQRAAGLYGSLIVDVAKGEKEPFHYDGEFNILLSDWWHESVHEQELGLSSKPFRWIGEPQTLLMNGRGQYNCSLAAHYSNSSSSQCMFRGNEQCAPQILRVRPNKIYRLRVTSSTSLASLNLQISNHKMVLVEADGNYLQPFAVDDMDIYSGESYSVLFTTDQNPSNNYWVSVSVRGRKPNTTQALTILNYHNTTSASKPPPSPPPVAPLWNNYTHSKLFTKKVLALMGNPNPPPTTHHRRIILLNTQNYIDGYTKWAINNISLVLPATPYLGSIKYNLNNAFDHKTPPANFPSNYDVMKQAQNPNSTYGSGVYMLSFNTTVDIILQNANALAVNTSEIHPWHLHGHDFWVLGYGEGKFSNENDEKSFNLKNPPYRNTAVVFPFGWTALRFVANNPGVWAFHCHIEPHLHMGMGVVFAEGVRRLGKIPNEVLTCGLTGKMFLNNKND | AT5G21105 | L-ascorbate oxidase | 66 % | 816 |
| CsAPX | MGKCYPTVSEEYKKAIDKAKRKLRGFIAEKNCAPLMLRLAWHSAGTYDVNSKTGGPFGTMRHKLEQGHEANNGLEIAVGLLEPLKEQFPILSYGDFYQLAGVVAVEITGGPDVPFHPGREDKPEPPIEGRLPDATKGCDHLRDVFVKHMGLSDKDIVVLSGGHTLGRCHKERSGFDGAWTTNPLIFDNTYFTELLTGEKEGLLQLQSDKALLDDPAFRPLVEKYAADEDAFFADYAEAHMKLSELGFAEA | APX1 | L-ascorbate peroxidase; Plays a key role in hydrogen peroxide removal. Constitutes a central component of the reactive oxygen gene network | 80 % | 434 |
| CsDHAR1 | MSTAKIHPSASALSTTIKHLTGTLQFSRTCTFPPNGLAHSRRTTHALRIRRSLTVSSSSVSDPLEVCVKASLTTPNRLGDCPFTQRVLLTLEEKHLPYDLKLVDFSKKPEWFLKVSPEGKVPVIKIDEKWIADSDVITQALEEKFPNPPLGTPPEKASVGSKIFSTFIGFLKSKDPNDGTEQALLSELVAFNDYLKENGPFVDGKRVSAADLSLGPKLYHLEISLGHYKNWSVPDSLPCVKSYMKAIFSMESFVKTRALPEDVIEGWRPKVMG | DHAR3 | dehydroascorbate reductase 1; Exhibits glutathione-dependent thiol transferase and dehydroascorbate (DHA) reductase activities. Key component of the ascorbate recycling system. Involved in the redox homeostasis, especially in scavenging of ROS under oxidative stresses | 71 % | 365 |
| CsDHAR2 | MALEVCAKAASGAPDILGDCPFTQRVLLTLEEKKIPYKIHLINISDKPQWFLEANPEGKVPVIKFDEEWISDSDVIVSHIEEKFPDPPL CHSPEVSSVGSKIFPSFVKFLKSKDPNDGSEQALLDELKALDEHLQAHGPYINGESVCAVDLGLAPKLYHLDVALGHFKGWKIPESLTHVHNYMKLLFSKECFEKTKAAKEHVVAGWAPKVNP | DHAR2 | dehydroascorbate reductase; Displays a dual function. As a soluble protein, exhibits glutathione-dependent thiol transferase and dehydroascorbate (DHA) reductase activities. Key component of the ascorbate recycling system. Involved in the redox homeostasis, especially in scavenging of ROS under oxidative stresses, subsequently to biotic or abiotic inducers. As a peripheral membrane protein, could also function as voltage-gated ion channel | 69 % | 306 |
| CsGGP | MMLRIKRVPTVVSNYQKEEAEEGARRPGGGCGRNCLQNCCILGAKLPLYAFKRVNKIVGEKGLIAHDYIEPSIAFLDSLLLGEWED  RMQRGLFRYDVTACETKVIPGEYGFIAQLNEGRHLKKRPTEFRVDKVLQPFDGNKFNFTKVGQEEVLFQFEASDEDNETQFFPNAPIDVVNSPSVVAINVSPIEYGHVLLIPWILECLPQRIDRVSFLLALYMAAEAGNPYFRLGYNSLGAFATINHLHFQAYYLAVPFPIEKAPIREITTLNGGVKISELLNYPVRGLVFEGGNTLEDLSNAVSDSCICLQDNNVPYNVLISESGKRIFLVPQCYAEKQALGEVSSELLDTQVNPAVWEISGHMVLKRKKDYEEASDKNAWRLLAEVSLSEERFQEVSALIFESITCIDDGNESVSQSFMGEPNATPQPLEEVDDLDKSS CHAMVPGKQECLVLH | VTC2 | vitamin C defective 2; Catalyzes a reaction of the Smirnoff-Wheeler pathway, the major route to ascorbate biosynthesis in plants. Acts as a phosphorylase rather than as a transferase. Uses preferentially GDP-L-galactose and GDP-D-glucose as substrates. Lower activity with GDP-L-fucose, very low activity with GDP-D-mannose, and no activity with UDP-D-glucose, UDP-D-galactose or ADP-D-glucose. Highly specific for inorganic phosphate as the guanylyl acceptor | 76 % | 636 |
| CsGME | MGSAGETNYGAYTYENLEREPYWPSEKLRVSITGAGGFIASHIARRLKSEGHYIIASDWKKNEHMTEDMFCHEFHLVDLRVMDNCLKVTTGVDHVFNLAADMGGMGFIQSNHSVIMYNNTMISFNMLEAARVNGVKRLFYASSACIYPEFKQLDTNVSLKESDAWPAEPQDAYGLEKLATEELCKHYTKDFGIECRVGRFHNIYGPFGTWKGGREKAPAAFCRKALTSTDKFEMWGDGLQTRSFTFIDECVEGVLRLTKSDFREPVNIGSDEMVSMNEMAEIVLSFENKKLPIHHIPGPEGVRGRNSDNTLIKEKLGWAPTMRLKDGLRITYFWIKEQIEKEKAKGINLSTYGSSKIVGTQAPVQLGSLRAADGKE | GME | GDP-mannose 3,5-epimerase; Catalyzes a reversible epimerization of GDP-D-mannose that precedes the committed step in the biosynthesis of vitamin C (L-ascorbate), resulting in the hydrolysis of the highly energetic glycosyl-pyrophosphoryl linkage. Able to catalyze 2 distinct epimerization reactions and can release both GDP-L-galactose and GDP-L-gulose from GDP-mannose | 90 % | 725 |
| CsGMP | MKALILVGGFGTRLRPLTLSVPKPLVDFANKPMILHQIEALKAIGVSEVVLAINYQPEVMLNFLKDFEAKLGIKITCSQETEPLGTAGPLALARDKLIDDSGEPFFVLNSDVISEYPLKEMIEFHKSHGGEASIMVTKVDEPSKYGVVVMEESTGQVERFVEKPKLFVGNKINAGIYLLNPSVLDRIELRPTSIEKEVFPKIAAQKQLYGMVLPGFWMDIGQPRDYITGLRLYLDSLRKKSPSKLSTGPHIVGNVLVDETSKIGEGCLIGPDVAIGPGCVVEAGVRLSRCTVMRGVRIKKHACISSSIIGWHSTVGQWARVENMTILGEDVHVCDEIYSNGGVVLPHKEIKSSILKPEIVM | CYT1 | CYTOKINESIS DEFECTIVE 1; Catalyzes a reaction of the Smirnoff-Wheeler pathway, the major route to ascorbate biosynthesis in plants. Plays an essential role in plant growth and development and cell-wall architecture. Provides GDP-mannose, used for cell wall carbohydrate biosynthesis, protein N-glycosylation, as well as for the biosynthesis of the antioxidant ascorbate | 93 % | 691 |
| CsGPP | MGRCLVSSTLTPLRFSQIRRSISLFNLPKLTLPSSSFAIPHLRRGFRDGFCKTLSFNSMPTRTLCTKAVLSEIPNQKKYSKVASESTGPISSNQLLGVVETAAKTGAEVVMDAVKKPQNIVYKGLTDLVTDTDKMSEVAILEVVTKNFKDHLILGEEGGLIGNSSSDYLWCIDPLDGTTNFAHCYPSFAVSVGVLYKGKPAAGAVVEFVGGPMCWNTRTFSAAAGGGAFCNGQKIQVSHTDKVEQSLLVTGFGYEHDDAWSTNIELFKEFTDVSRGVRRLGAAAVDMCHVALGIVEAYWEYRLKPWDMAAGVLIVEEAGGVVSCMDGGKYSVFDRSVLVSNGVLHDKLLERIGPPTDKLKNKGIDFSLWFKPENYHTDC | IMPL1 | myo-inositol monophosphatase like 1; Phosphatase acting preferentially on D-myoinositol 1- phosphate (D-Ins 1-P) | 74 % | 558 |
| CsGR | MATSLSAPKLSTTFSSSPTLQTLRRTLPISLSHPFLSPHSPSSPLFLSPRRLRPSFSHHHHRRFSARAESDNGAEPRHYDFDLFTVGAGSGGVRASRFAANFGASVAVCELPFATISSETSGGVGGTCVLRGCVPKKLLVFASKYSHEFEESHGFGWKYETEPKHDWSTLMANKNAELQRLIGIYKNILKNAGVTLIEGRGKIVDPHTVDVDGKLYSARHILVAVGGRPSIPEIPGSEYAIDSDAALDLPSRPEKIAIVGGGYIALEFAGIFNGLRSDVHVFIRQKKVLRGFDEEVRDFVAEQMSLRGIEFHTEESPQAIIKSADGSLSLKTNRGTVEGFSHIMFATGRKPNTKNLGLEKVGVKMAKNGAIEVDEFSCTSVPSIWAVGDVTDRVNLTPVALMEGGALAKTLFRNEPTKPDHRAIPSAVFSQPPIGQVGLTEEQAVNEYGDVDIFTANLRPLKATLSGLPDRIFMKLIVCAKTNKVLGLHMCGEDSPEIVQGFAVAVKAGLTKAELDATVGIHPTSAEEFVTMRTPTRKIRNGPPPEGTMDSVVKAAAGV | GR | Glutathione reductase; Maintains high levels of reduced glutathione in the chloroplast | 77 % | 877 |
| CsGalDH | MANLELRSLGNTGLKLSSVGFGASPLGNVFGPVSEDDAFASVRDAFRLGINFFDTSPYYGGTVSEKVLGKALKAMGMPRNEYIVSTKCGRYVDGFDFSAERVTRSIDESLDRLQLDYVDILQCHDIEFGSLDQIVNETIPALQKLKEAGKIRFIGITGLPLGVFTYVLDRVPPGTVDVILSYCHYSINDSTLEDLLPYLKSKGVGVISASPLSMGLLTERGPPEWHPALPELKAACQAAAAYCKENGKNISKLAMQYSLSNKDISSVLVGMNSVKQVEENVAAAKELAMFGKDEKAVSEIEEILKPVMNQTWPSGIQQS | AT4G33670 | D-threo-aldose 1-dehydrogenase; Catalyzes the oxidation of L-galactose to L-galactono- 1,4-lactone in the presence of NAD(+). Uses NAD(+) as a hydrogen acceptor much more efficiently than NADP(+) | 78 % | 534 |
| CsGalLDH | MVNLALLDRVLEVDKEKKTVRVEAGIRVQQLVDGIKDYGLTLQNFASIREQQIGGIVQVGAHGTGARLPPIDEQVVSMKLVTPAKGTIEVSKEKDPELFYLARCGLGALGVVAEVTLQCVERQELVEHTFVSNTEEIKKNHKKFLSENKHVKYLYIPYTDTVVVVRCNPVSKWKGPPKFKPKYSHDEAMQNVRDLYQESLKKYRRAVTTTESVDNNEQDINELSFTELRDKLLALDPLNKNHIIKVNQAEAEFWRKSEGYRVGWSDEILGFDCGGQQWVSETCFPAGTLSKPSMKDLEYIEELMQLIDKEAIPAPAPIEQRWTARSKSLMSPASSTADDDIFSWVGIIMYLPTMDARQRKEITDEFFHYRHLSQSQLWDRYSAYEHWAKIEVPKDKDELATLQARLRKRFPVDAYNKARRELDPNHILSNNMLEKLFPQSDII | GLDH | L-galactono-1,4-lactone dehydrogenase; Involved in the biosynthesis of ascorbic acid. Required for the accumulation of respiratory complex I. Uses L-galactono- 1,4-lactone and L-gulono-1,4-lactone as substrates, but not D- galactono-1,4-lactone, D-gulono-1,4-lactone, L-mannono-1,4-lactone or D-galactonic acid. Also active with phenazine methosulfate and 1,4-benzoquinone as electron acceptors | 84 % | 780 |
| CsGalUR | MMKSTPDMLLCSSTGTDHKQIPLLGFGTAVYPFLSSETMKQSILQAIKFGYRHFDSAALYRSEQPLGEAIADAIRLGFIQSRQDLFITSKLWCSDAHRDHVLPAIQKSLKNLGLEYLDLYLVHWPVSSKPGKYELPVNKQELLPLDFKSVWEAMEECQSLGLTKSIGVSNFSCKKLQLLLSTAKIPPAVNQVEMNPLWQQKKLRDFCEKNGIHVTAYSPLGAKGTLWGSNQVMECEVLKQIAKARGKSVAQVCLRWVYEQGVSVLVKSFNEERIKENLHIFDWELSAQDSEMINQILQRKGFPGVEFISDVGPYKSLQDLWDGEIAX | AT1G59960 | putative Aldo/keto reductase | 55 % | 399 |
| CsMDHAR | MAEKTFKYVILGGGVSAGYAAREFAKQGVKPGELAIISKEGVAPYERPALSKAYLFPESPARLPGFHVCVGSGGERLLPEWYAQKGIALILNTEIVKADLATKTLVSAAGETFNYHFLIIATGSSVIRLTDFGVQGADAKNIYYLREIDDADKLVEAIQAKKNGKVVIVGGGYIGLELSAVMKLNNLDVNMVYPEPWCMPRLFTAGIAAFYEGYYANKGIKIIKGTVAVGFTADANGEVKEVKLKDGRVLEADIVVVGVGGRPLTTLFKGQVEEEKGGIETDSFFKTSVPHVYAVGDVATFPMKIYNEMRRVEHVDHARKSAEHAVKAIFASTEGKSIEEYDYLPFFYSRSFNLSWQFYGDNVGDTVLFGDNSPTSENPKFGSYWIKDGKVVGAFLESGTPEENKAIAKVARVQPPVESLDLLAKDGLTFACKI | MDAR1 | Monodehydroascorbate reductase 1; Catalyzes the conversion of monodehydroascorbate to ascorbate, oxidizing NADH in the process (By similarity) | 72 % | 663 |
| CsMIOX | MTILIDQPDFGVEAHEEHEEQKIPNNENELVLDGGFVVPEINSFGQNFRDYDAESLRQQGVEDFYRVNHINQTFDFVKKMREKYGKVDKVEMSIWECCELLNDVVDESDPDLDEPQIEHLLQTAEAIRKDYPNEDWLHLTGLIHDLGKVLLLPSFGELPQWAVVGDTFPVGCAFDESIVHHKYFKENADYENPAYNTKFGIYSEGCGLNNVMMSWGHDDYMYLVAKENHTTLPSAGLFIIRYHSFYALHKSGAYKHLMNEEDIENLKWLQIFNKYDLYSKSKVRIDVEKVKPYYLSLIKKYFPEKLRW | MIOX1 | Inositol oxygenase 1; Involved in the biosynthesis of UDP-glucuronic acid (UDP-GlcA), providing nucleotide sugars for cell-wall polymers. May be also involved in plant ascorbate biosynthesis | 78 % | 510 |
| CsPGI1 | MASSALICDKEQWKDLKAHVDDIKKTHLRELMSDSERCKSMMAEFDGILLDYSRQCATLETMNKLFKLAEAACLKEKINKMFNGERINSTENRSVLHVALRAPRDAVINSNGKNVVPDVWNVLDKIRDFSERVRSGSWVGATGKALTNVIAIGIGGSFLGPLFVHTALQTDPEAIECARGRHLRFLANVDPIDVARNITGLNPETTLVVVVSKTFTTAETMLNARTLREWISSALGPEAVSKHMVAVSTNLTLVEKFGIDPKNAFAFWDWVGGRYSVCSAVGVFPLSLQYGFLVVEKFLKGASSIDQHFYSAPFDQNIPVLLGLLSVWNVSFLGYPARAILPYSQALEKLAPHIQQVSMESNGKGVSIDGVALPYEAGEIDFGEPGTNGQHSFYQLIHQGRVIPCDFIGIVKSQQPVYLKGEVVSNHDELMSNFFAQPDALAYGKTPQQLQSENVSQHLIPHKTFSGNRPSLSLLLPSLSAYNVGQLLAFYEHRIAVEGFIWGINSFDQWGVELGKSLASQVRKQLHASRKKGEPVEGFNFSTTTMLNRYLQESSDVPPDPSTLLPHM | AT5G42740 | Glucose-6-phosphate isomerase | 84 % | 977 |
| CsPGI2 | MASISGLSSSSSTLKPEKFTPKSTPSSLPSRDSIAFPNRSKFFDRASTLSPQSVARDIPASLSSTNDGLSKEKKKGLLKNPRELWRRYVDWLYQHKDLGLYLDVSRIGFTDEFVGEMEPKFQAAFKAMEELEKGSIANPDEGRMVGHYWLRNPKLAPKSILRLQIENTLEAVRKFADDVVSGKIKPPSSPEGRFTHVLSVGIGGSALGPQFVAEALAPDNPPLKIRFIDNTDPAGIDHQIAQLGPELASTLVIVISKSGGTPETRNGLLEVQKAFRDSGLDFAKQGVAITQENSLLDNTARIEGWVARFPMFDWVGGRTSEMSAVGLLPAALQGIDIKEMLAGASLMDEANRTTVVRNNPAALLALCWYWASDGVGSKDMVVLPYKDSLLLFSRYLQQLVMESLGKEFDLEGNRLWRSAHRTPKQPRSFG | PGI1 | Phosphoglucose isomerase 1; Promotes the synthesis of starch in leaves | 77 % | 658 |
| CsPMI | MLMESNGSSNRRRTLQRLRCSVKNYDWGRIGCESRVSRLFSRNSGLHIEEGKPYAEFWMGTHESGPSFVLENGGLSLNSWIAKNPNVLGDKVVQKWGVNLPFLFKVLSVSKALSIQAHPDKEFAGFLHKTRPDVFKDDNHKPEMALALTEFEALCGFISLKELKDVLQNVPEIVEVVGSVYANQVLTINHEDGEEKVKSVLRSIFTQLMSASKDVISKALSNLKSRLNQVRQLTDKEQLVLRLEKQYPADVGVLAAFLFNYVKLKPGEALYLGANELHAYLHGECIECMATSDNVVRAGLTPKSRDAQILCSMLTYKQGFPEILQGVPLNPYTRRYLPPFDEFEVDRCILPQGASVVFPAVPGPSVFVIMEGEGTMHASSFEDVVREGDVLFTPANTDISVRTASELHLYRAGVNSRFFQAS | MEE31 | Mannose-6-phosphate isomerase; Involved in the synthesis of the GDP-mannose and dolichol-phosphate-mannose required for a number of critical mannosyl transfer reactions. Involved in the ascorbic acid (AsA) biosynthesis. Required during the endosperm development | 63 % | 539 |
| CsPMM | MFLRCRSASVSAFTLYSTLLASFSSPNLPQKILYTEMAARKPGLIALFDVDGTLTAPRKVVTPDMLKFMQELRKVVTVGVVGGSDLVKISEQLGNSVINDYDYVFAENGLVAYKDGKLIGTQSLKSYLGEEKLKEFINFTLHYIADVDIPIKRGTFIEFRSGMLNVSPIGRNCSQEERDEFEKYDKVLNIRPKMVSVLREKFAHLNLTFSIGGQISFDVFPQGWDKTYCLRYVDDFHEIHFFGDKTYKGGNDHEIYESERTMGHTVTSPEDTVKQCTALFLSKQV | PMM | Phosphomannomutase; Involved in ascorbic acid biosynthesis and in the synthesis of the GDP-mannose and dolichol-phosphate-mannose required for a number of critical mannosyl transfer reactions | 80 % | 427 |
