# Supplementary figures and images for: Differentially expressed protein and gene analysis revealed the effects of temperature on changes in ascorbic acid metabolism in harvested tea leaves
Source: Hortic Res. 2018 Oct 1;5:65. doi: 10.1038/s41438-018-0070-x (PMC6165846; doi:10.1038/s41438-018-0070-x)

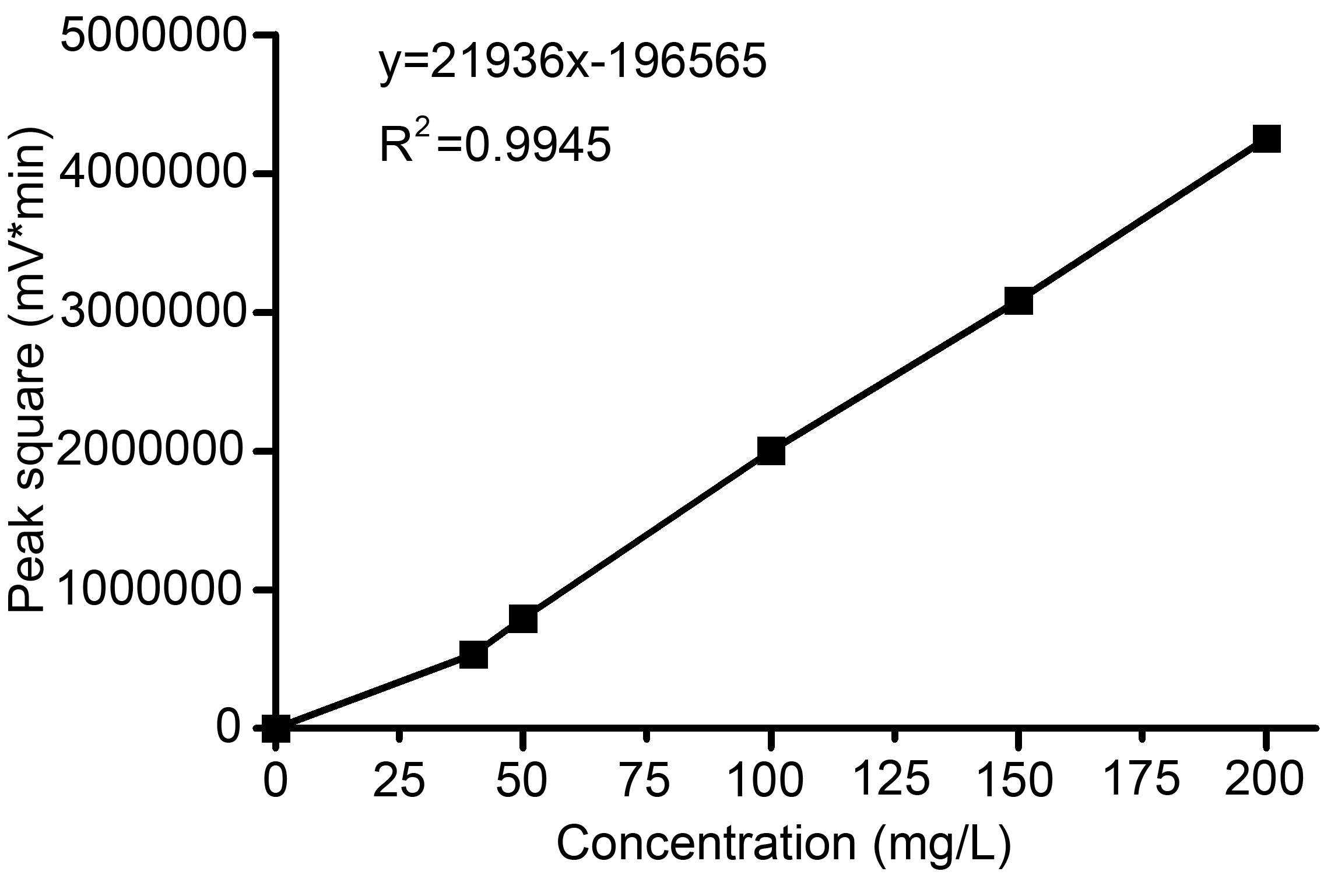

Supplement: Supplementary file 5 — Figure S1 [file 41438_2018_70_MOESM5_ESM.jpg]
